# Dynamic RNA profiles in the small intestinal epithelia of cats after* Toxoplasma gondii* infection

**DOI:** 10.1186/s40249-023-01121-z

**Published:** 2023-07-25

**Authors:** Bintao Zhai, Shi-Chen Xie, Jiyu Zhang, Jun-Jun He, Xing-Quan Zhu

**Affiliations:** 1 Key Laboratory of Veterinary Pharmaceutical Development, Ministry of Agriculture and Rural Affairs, Lanzhou Institute of Husbandry and Pharmaceutical Sciences, Chinese Academy of Agricultural Sciences, Lanzhou, 730050 Gansu People’s Republic of China; 2 State Key Laboratory for Animal Disease Control and Prevention, Key Laboratory of Veterinary Parasitology of Gansu Province, Lanzhou Veterinary Research Institute, Chinese Academy of Agricultural Sciences, Lanzhou, 730046 Gansu People’s Republic of China; 3 Laboratory of Parasitic Diseases, College of Veterinary Medicine, Shanxi Agricultural University, Taigu, 030801 Shanxi People’s Republic of China; 4 Research Center for Parasites & Vectors, College of Veterinary Medicine, Hunan Agricultural University, Changsha, 410128 Hunan People’s Republic of China; 5 Key Laboratory of Veterinary Public Health of Yunnan Province, College of Veterinary Medicine, Yunnan Agricultural University, Kunming, 650201 Yunnan People’s Republic of China

**Keywords:** *Toxoplasma gondii*, Definitive host, Cat, Small intestinal epithelia, Long non-coding RNA, Circular RNA, mRNA

## Abstract

**Background:**

Felids are the only definitive hosts of *Toxoplasma gondii.* However, the biological features of the feline small intestine following *T. gondii* infection are poorly understood. We investigated the changes in the expression of RNAs (including mRNAs, long non-coding RNAs and circular RNAs) in the small intestinal epithelia of cats following *T. gondii* infection to improve our understanding of the life cycle of *T. gondii* and cat responses to *T. gondii* infection.

**Methods:**

Fifteen cats were randomly assigned to five groups, and the infection groups were inoculated with 600 tissue cysts of the *T. gondii* Pru strain by gavage. The small intestinal epithelia of cats were collected at 6, 10, 14, and 30 days post infection (DPI). Using high-throughput RNA sequencing (RNA-seq), we investigated the changes in RNA expression. The expression levels of differentially expressed (DE) genes and non-coding RNAs (ncRNAs) identified by RNA-seq were validated by quantitative reverse transcription PCR (qRT-PCR). Differential expression was determined using the DESeq R package.

**Results:**

In total, 207 annotated lncRNAs, 20,552 novel lncRNAs, 3342 novel circRNAs and 19,409 mRNAs were identified. Among these, 70 to 344 DE mRNAs, lncRNAs and circRNAs were detected, and the post-cleavage binding sites between 725 ncRNAs and 2082 miRNAs were predicted. Using the co-location method, we predicted that a total of 235 lncRNAs target 1044 protein-coding genes, while the results of co-expression analysis revealed that 174 lncRNAs target 2097 mRNAs. Pathway enrichment analyses of the genes targeted by ncRNAs suggested that most ncRNAs were significantly enriched in immune or diseases-related pathways. NcRNA regulatory networks revealed that a single ncRNA could be directly or indirectly regulated by multiple genes or ncRNAs that could influence the immune response of cats. Co-expression analysis showed that 242 circRNAs, mainly involved in immune responses, were significantly associated with *T. gondii* infection. In contrast, 1352 protein coding RNAs, mainly involved in nucleic acid process/repair pathways or oocyte development pathways, were negatively associated with *T. gondii* infection.

**Conclusions:**

This study is the first to reveal the expression profiles of circRNAs, lncRNAs and mRNAs in the cat small intestine following *T. gondii* infection and will facilitate the elucidation of the role of ncRNAs in the pathogenesis of *T. gondii* infection in its definitive host, thereby facilitating the development of novel intervention strategies against *T. gondii* infection in humans and animals.

**Graphical Abstract:**

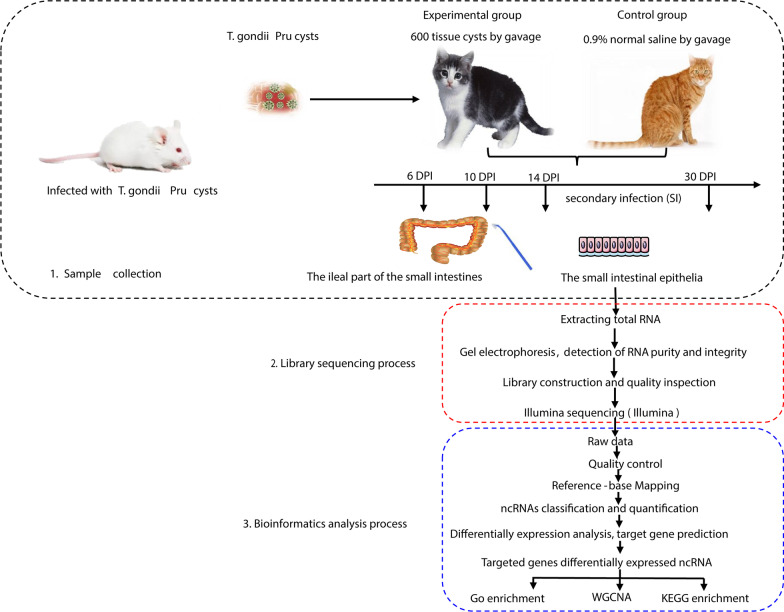

**Supplementary Information:**

The online version contains supplementary material available at 10.1186/s40249-023-01121-z.

## Background

Toxoplasmosis, a cosmopolitan zoonosis in humans and warm-blooded animals, is caused by infection with *Toxoplasma gondii* [1, 2], which has a wide range of intermediate hosts, including all warm-blooded vertebrates. Felids are the only known definitive hosts of *T. gondii*. Sexual reproduction of *T. gondii* is triggered only in the intestinal epithelial cells of felid animals, and more than one million oocysts can be produced and shed following primary infection [3]. Cats develop immunity against re-infection by oocysts following rechallenge with homologous or heterologous *T. gondii* [4]. Generally, ingestion of sporulated oocysts or tissue cysts leads to mild clinical symptoms, such as fever in humans; however, infection with *T. gondii* can be fatal to the foetus [5] and patients with acquired immunodeficiency syndrome (AIDS) [2, 6]. *T. gondii* infections are also linked to several diseases of the central nervous system (CNS) and muscles [7, 8]. Epidemiological surveys have revealed that cats play an important role in the spread of *T. gondii*. Therefore, understanding the interplay between cats and *T. gondii* is critical to prevent the spread of toxoplasmosis.

Transcriptomic technologies are widely used to study host–pathogen interactions because they can provide important data on the interplay between host and pathogens [9]. However, the intracellular RNA networks are complex [10]. More than 90% of the genome can be transcribed into RNA [11, 12], whereas less than 2% can be translated into proteins. Several studies have revealed the differential expression of *T. gondii* proteins at different developmental stages [13, 14]; however, only a limited number of studies have been performed on proteomic changes in cat organs after infection with *T. gondii* [15]. Non-coding RNAs (ncRNAs), including long non-coding RNAs (lncRNAs), circular RNAs (circRNAs) and microRNAs (miRNAs), are important components that regulate protein production and the corresponding cellular biological processes [16]. LncRNAs are defined as RNA molecules with a length greater than 200 nt that have no protein-coding ability. However, previous studies have demonstrated that the secondary structure of lncRNAs is conserved and that lncRNAs regulate various biological processes by interacting with proteins, DNA, and RNA [17–19]. LncRNAs are known to regulate biological functions at the epigenetic, transcriptional, and post-transcriptional levels and play a role in X-chromosome silencing, genomic imprinting, chromatin modification and transcriptional activation or repression [20–22]. In addition, lncRNAs are closely associated with various diseases [23]. For example, a novel lncRNA, NONSHAT022487, was reported to regulate immune responses by suppressing the expression of immune-related molecules, including UNC93B1mRNA [24]. MiRNAs are single-stranded non-coding RNAs with lengths of 19–25 nt. They function as negative regulators of gene expression at the posttranscriptional level by binding with the 3′‐untranslated region (3′‐UTR) of target mRNAs [25] and subsequently influence various cellular biological processes, including proliferation, differentiation, apoptosis and migration [26, 27]. A recent study reported that *T. gondii* infection modulates the expression of immune-related cytokines and C-type lectins by altering miRNA expression [28]. For instance, the inhibition of miR-20a function promotes apoptosis in human macrophages induced by *T. gondii* infection [29]. CircRNAs are RNA molecules with closed-loop structures. These molecules are stably expressed and different from the traditional linear RNAs with distinct 5′ and 3′ ends [30]. Recent studies demonstrated that circRNAs are rich in miRNA-binding sites and act as miRNA sponges. CircRNAs abolish the inhibitory effects of miRNAs on target genes and increase the expression levels of miRNA target genes via a competing endogenous RNA (ceRNA) mechanism [31, 32]. CircRNAs also play an important regulatory role in disease development by interacting disease-associated miRNAs [33]. Unfortunately, limited information is available on how circRNAs respond to *T. gondii* infection in their definitive host, which limits efforts to prevent or control *T. gondii*.

*Toxoplasma gondii* was discovered more than one hundred years ago. However, the mechanism of sexual development of *T. gondii* remains to be elucidated, and the role of miRNAs, lncRNAs, and circRNAs in sexual development of *T. gondii* has not been studied. Determining the roles of these non-coding RNAs in the sexual development of *T. gondii* is of great significance for the development of intervention strategies against *T. gondii* infection in humans and animals.

Interestingly, felids show strong immunity against the development of oocysts following primary infection with *T. gondii*. Previous transcriptomic studies have shown that *T. gondii* infection of the definitive host causes immune defense responses in various organs of cats [9, 34]. However, triggering of the immune response is common in pathogen infections, and the pathway that contributes to a strong immune defense remains unknown.

Therefore, studying the molecular changes in the small intestine of cats caused by primary or secondary *T. gondii* infections is of great public health significance. The intestinal ceRNA network of cats infected with *T. gondii* is yet to be investigated. This study is the first to employ high-throughput RNA sequencing technology to investigate the expression profiles of mRNAs, lncRNAs, and circRNAs associated with primary or secondary *T. gondii* infections in the feline small intestine.

## Methods

### Ethics statement

The animal experimental protocols were approved by the Animal Ethics Committee of Lanzhou Veterinary Research Institute (LVRI), Chinese Academy of Agricultural Sciences (CAAS) (Protocol Permit Number: LVRIAEC-2018-006). Efforts were made to minimise the suffering of cats and reduce the number of animals used in the experiment.

### Parasite strain, induction of infection, and sample collection

The *T. gondii* Prugniuad (Pru) strain (genotype II) was maintained in our laboratory and passaged in Kunming mice. Female Kunming mice, aged 6–8 weeks, were purchased from the Laboratory Animal Center of Lanzhou Veterinary Research Institute, Chinese Academy of Agricultural Sciences. The mice had *libitum* access to sterile food and water, and were raised in a spacious cage at ± 25 ℃. The infected mice were humanely euthanised to collect the *T. gondii* tissue cysts from the brain, which were homogenised and counted microscopically. Fifteen domestic cats (Chinese Li Hua breed, 7–9 months old) were purchased from a local breeder and raised in a spacious cage at ± 25 ℃ with sufficient food and water. Sera were tested using an enzyme-linked immunosorbent assay (ELISA) (Enzo, Hubei, China) to ensure that they were free from infections with the four feline viruses (feline immunodeficiency virus, feline leukemia virus, feline calicivirus, and feline parvovirus). A modified agglutination test (MAT, cut-off 1:25) was performed to confirm that the cats were free from *T. gondii* infections [35]. The cats were kept under laboratory conditions for one month to build a similar intestinal flora. The cats were randomly assigned to five groups: one control group, three primary infection groups, and one secondary infection group, with three replicates in each group. The cats in the control group were inoculated with 0.9% normal saline by gavage, whereas the cats in the infection groups, including the three primary infection groups and one secondary infection (SI) group, were inoculated with 600 tissue cysts of the *T. gondii* Pru strain by gavage. The cats in the secondary infection group were reinoculated with 600 cysts of Pru strain two weeks after they stopped shedding oocysts. All cats were humanely euthanized for tissue samples collection. The ileal sections of the small intestine of the control and primary infection groups were harvested at 6, 10, and 14 days post infection (DPI). The small intestinal tissues of the secondary infection group were harvested at 30 DPI, following the rechallenge with 600 cysts at 27 DPI. The collected intestinal tissues were washed thoroughly with PBS to remove the intestinal contents. The small intestinal epithelia were collected using a cell lifter, frozen in liquid nitrogen, and stored at − 80 °C until use. The remaining small intestinal tissues were fixed and stained with hematoxylin and eosin (H&E).

### Detection of *T. gondii* infection in small intestinal epithelia

To monitor the shedding of *T. gondii* oocysts, feline faecal samples were collected daily and examined using the saturated sucrose flotation method [36]. Genomic DNA was extracted from the small intestinal epithelia using a TIANamp Genomic DNA kit (TianGen™, Beijing, China) according to the manufacturer’s instructions. A PCR assay was used to detect *T. gondii* infection as previously described [37]. The PCR had a total volume of 50 μl, containing 25 μl 2 × Phanta Max Buffer (Vazyme version 9.1), 1 μl dNTP Mix (10 mmol/L), 2 μl of each primer (10 μmol/L), 1 μl Phanta Max Super-fidelity DNA polymerase, 1 μl template DNA, and 18 μl distilled water. The amplification conditions were as follows: denaturation at 95 °C for 5 min followed by 35 cycles at 95 °C for 10 s, annealing at 60 °C for 10 s, and extension at 72 °C for 20 s. A positive control (DNA from *T. gondii* laboratory standard Pru strain) and negative control (ddH_2_O) were included in each PCR run. The PCR amplification products were analysed by electrophoresis on 1% agarose gels stained with Sparkred nucleic acid gel stain (AJ0210, Sparkred, Changsha, China). Positively amplified fragments were sequenced by Tsingke Biotechnology Co., Ltd. The obtained sequences were analysed using a BLASTn search (http://blast.ncbi.nlm.nih.gov/Blast.cgi) against GenBank and the using Clustal W method in the MegAlign software (DNAStar, Madison, WI).

### Library preparation for RNA sequencing

The total RNA was separately extracted from the small intestinal samples, and the RNA quality was assessed using the RNA Nano 6000 Assay Kit of the Bioanalyzer 2100 system (Agilent Technologies, CA, USA) and Qubit® RNA Assay Kit in Qubit® 2.0 Flurometer (Life Technologies, CA, USA). Twenty ng (circRNA: 5 μg, small RNA: 3 μg) of RNA from each sample was used as the input material for preparing the RNA library. First, ribosomal RNA was removed using the Epicentre Ribo-zero™ rRNA Removal Kit (Epicentre, Wisconsin, USA), and the rRNA-free residue was cleaned by ethanol precipitation. Subsequently, the NEBNext® Ultra™ Directional RNA Library Prep Kit for Illumina® (NEB, MA, USA) was used to generate sequencing libraries, according to the manufacturer’s instructions. Briefly, fragmentation was performed using divalent cations at elevated temperatures in NEBNext First Strand Synthesis Reaction Buffer (5×). For small RNA, NEB 3′ SR Adaptor was directly, and specifically ligated to 3′ end of miRNA, siRNA and piRNA. After the 3′ ligation reaction, the SR RT primer was hybridised to an excess of 3′ SR adaptor, and the single-stranded DNA adaptor was transformed into a double-stranded DNA molecule. 5′ ends adapter was ligated to 5′ ends of miRNAs, siRNA and piRNA. First-strand cDNA was synthesised using random hexamer primers and M-MuLV reverse transcriptase (RNase H). Second-strand cDNA was synthesised using DNA polymerase I and RNase H. After adenylation of 3′ ends of DNA fragments, NEBNext adaptor with hairpin loop structure were ligated to prepare for hybridisation. First- and second-strand cDNA synthesis was subsequently performed, and an AMPure XP system (Beckman Coulter, Beverly, USA) was used to purify the lncRNA/circRNA fragments from the library to select cDNA fragments with lengths of 150–200 bp. Finally, library quality was assessed using an Agilent Bioanalyzer 2100 system. The index-coded samples were clustered using the TruSeq PE Cluster Kit v3-cBot-HS (Illumina, CA, USA), according to the manufacturer’s instructions. Following cluster generation, the lncRNA libraries were sequenced using an Illumina Hiseq 2500 platform, and 125 bp paired-end reads were generated. CircRNA libraries were sequenced using an Illumina Hiseq 4000 platform, and 150 bp paired-end reads were generated. Small RNA libraries were sequenced on an Illumina Hiseq 2500/2000 platform and 50 bp single-end reads were generated.

### Quality control

Raw data (raw reads) in FASTQ format were initially processed using in-house Perl scripts (small RNAs through custom Perl and Python scripts). Clean data (clean reads) were obtained by removing the reads containing adapter sequences, poly-*N*, and low-quality reads from the raw data. Sequencing quality (Q20 and Q30) and GC content of the clean data were calculated. Clean data of high sequencing quality were used for further analyses.

### Transcriptomic analysis

The reference genome and gene annotation files were retrieved from the Ensembl genome database (version: *Felis catus* 9.0) and ToxoDB (https://toxodb.org/toxo/app/). The lncRNA reads obtained from the bacteria were filtered and the clean paired-end reads were aligned to the reference genome using HISAT2 (v2.0.4, http://daehwankimlab.github.io/hisat2/), which was run with '-rna-strandness RF', and other parameters were set as per default. The mapped lncRNA reads for each sample were assembled with StringTie (v1.3.3, https://github.com/gpertea/stringtie) using a reference-based approach [38]. StringTie uses a novel network flow algorithm and an optional de novo assembly step to assemble and quantify full-length transcripts representing multiple splice variants for each gene locus. The expression levels of lncRNAs and coding transcripts in each sample were evaluated from fragments per kilobase of transcript per million mapped reads (FPKMs) of lncRNAs and coding transcripts in each sample [39]. The circRNA index of the reference genome was built using Bowtie2 (v2.2.8, https://bowtie-bio.sourceforge.net/bowtie2/manual.shtml), and paired-end clean reads were aligned to the reference genome using Bowtie2 [40]. Small RNA tags were mapped to the reference sequence using Bowtie [41] without mismatches to analyse their expression and distribution on the reference. Mapped small RNA tags were used to identify known miRNAs. miRBase 20.0 was used as a reference, and the modified software mirdeep2 [42] and srna-tools-cli were used to obtain potential miRNAs and draw their secondary structures. Customised scripts were used to obtain the miRNA counts and base bias at the first position of the identified miRNA with a certain length and position of all identified miRNAs, respectively. In our analysis pipeline, miRNAs which may have base edits were detected by aligning all sRNA tags to mature miRNAs, allowing one mismatch. For known miRNA, miFam.dat (http://www.mirbase.org/ftp.shtml) was used to search for families, and a novel miRNA precursor was submitted to Rfam (http://rfam.sanger.ac.uk/search/) to identify Rfam families.

### NcRNA coding potential and regulatory analysis

CNCI [43], CPC2 [44], and Pfam-Scan [45] were used to distinguish between mRNAs and lncRNAs. PhyloFit (Phast, v1.3, https://github.com/gpertea/stringtie) was used to compute the phylogenetic models of the conserved and non-conserved transcripts among species, and phylop was used to compute a set of conservation scores for the lncRNAs and mRNAs. To explore the functions of the lncRNAs, their biological functions were predicted based on the protein-coding genes with which they were co-localised or co-expressed. The cis role is to act on neighbouring target genes, and the 10 k/100 k region upstream and downstream of the lncRNAs was used to predict the cis-target gene. The trans role is to identify each other by the expression level, the expressed correlation between lncRNAs and mRNA with R function "cor.test", and co-expression analysis method was used along with Pearson’s correlation coefficient (Pearson’s correlation > 0.95 or <  −0.95). CircRNAs were detected and identified using the find_circ script [46] and CIRI2 tool [47], and the raw counts were first normalised using TPM (libsize is the sum of circRNA read counts) [48]. The miRNA target sites in the circRNAs were predicted using miRanda (version 3.3a) [49]. The target genes of miRNA were predicted using miRanda. The miRNA expression levels were estimated as transcript per million (TPM) using the following criteria [48].

### Differential expression analysis

Differential expression analyses of the two conditions/groups were performed using the DESeq R package (https://www.bioconductor.org/packages/2.13/bioc/html/DESeq.html). The resulting *P* values were adjusted using Benjamini and Hochberg’s approach to control the false discovery rate [50]. The lncRNA and mRNA screening thresholds were *Q* value < 0.05, and the circRNA screening conditions were *P* value < 0.05. Hierarchical clustering of the samples was performed for obtaining an overview of the expression profile characteristics based on the FPKMs of the differentially expressed (DE) ncRNAs using the pheatmap R package in (https://github.com/raivokolde/pheatmap).

### Functional enrichment, network visualization, and WGCNA

The KEGG Orthology-Based Annotation System (KOBAS) 3.0 (http://kobas.cbi.pku.edu.cn/index.php) was used for functional and pathway annotation of the differentially expressed genes (DEGs) or lnc/circRNA pairs, and *P*-adjust/*Q-*value < 0.05 was considered to be significantly enriched. NcRNA regulatory networks were constructed to identify the roles of ncRNAs and their interactions with mRNAs during *T. gondii* infection of cat small intestinal epithelia. The mechanism of interaction between lncRNAs and mRNAs predicts the biological functions of lncRNAs through their co-location and co-expression with protein-coding genes. MicroRNA target sites in the exons of the circRNA were identified using miRanda (version 3.3a). The miRNA targets were predicted based on the conserved target sites and free energy of formation. The lncRNA–miRNA–mRNA and circRNA-miRNA-mRNA interactions were visualised using Cytoscape software (version 3.9.1, https://cytoscape.org/). The mRNAs, miRNAs, and ncRNAs that were significantly associated with *T. gondii* infection in the small intestinal epithelia of cats were identified using the weighted gene co-expression network analysis (WGCNA) package (v1.47) in R to systematically identify the gene sets associated with *T. gondii* infection at 6, 10, 14, and 30 (SI) DPI. The FPKM values of the transcripts were used as raw input data. RNAs with *P* value of gene significance of infection (*P*.GS.Infeciton) < 0.05, and belonging to the gene expression module that was significantly associated with *T. gondii* infection, were identified as the RNAs that were significantly associated with *T. gondii* infection.

### Verification of RNA-seq results by quantitative reverse transcription PCR (qRT-PCR)

The RNA-seq results were confirmed using qRT-PCR. Total RNA was extracted and reverse transcribed to single strand cDNA using a PrimeScript™ RT reagent kit (Takara, Dalian, China). A total of 28 genes were randomly selected for validation by qRT-PCR, and β-actin was selected as an endogenous reference gene. qRT-PCR was performed on a BIO-CFX96 system (Bio-Rad, CA, USA) using SYBR Green GoTaq® PCR Master Mix (Promega, Beijing, China), according to the manufacturer’s instructions. qRT-PCR was performed in triplicate, and the primers used are listed in Table 1. The following conditions were used for qRT-PCR: denaturation at 95 °C for 2 min followed by 40 cycles at 95 °C for 10 s, annealing at 58 °C for 15 s, and extension at 72 °C for 40 s. Melting curve analysis was performed at a temperature ranged of 72 °C to 95 °C for ensuring that the specific product was amplified in each reaction. The 2^−ΔΔCT^ method was used for normalising the relative change in gene expression.

**Table 1:** Primer sequences used for qRT-PCR

NCBI accession number	Primer F	Primer R	Gene name or Remark
LncRNA			
XLOC_122170	GAGAGAGAGGGAGACACAGAAT	GGTTAAGCGTCCGACTTCAG	
XLOC_021327	AGGGAAGTCTGCCACTATCT	GTTCACCATCGGCAAAGAATG	
XLOC_122162	GACGTGTGCGTGTCCTC	GAGCGTGACGGACTGAC	
XLOC_433161	AGGCGATTGATCGGCAAG	AGGACACATTGATCATCGACAC	
XLOC_119975	CATAGAAGCGGGAGCTATGTT	CTCCAGTGAACCCACTGAC	
XLOC_494167	ATGGAATCGGAGTCAGTGTTG	GAGTCTTTCTGGCCCTGTATG	
XLOC_123143	AGACGGGCATTGTCAATCTG	CAGCCACTACTTCAAGACCATC	
XLOC_429073	TCTCACGTCACTCAGGATCT	GAAGAATCACGAGGAGGAAGTC	
mRNA			
ENSFCAG00000030029	CCTTCTCACCCTTACGTTCATC	CAACTGCTAGTACCCTCCATAAC	
ENSFCAG00000025949	CCGTAAGCCCTTGGTCATAAT	TTGACGGCTACAACCTGAAG	HSPD1
ENSFCAG00000042651	GCCAGGACTTGGTCCTATTAAG	TACCACGACCGTAGGAGTTAG	KRT19
ENSFCAG00000040725	GAAGGAGCTGAAGGAGCTTATC	TTGTTCCGGTCAAGGTCATC	S100A6
ENSFCAG00000006483	TACCTCTACCTGTCCTCTTGAC	CTTCCTCCTCCTCCTCCTTATT	PIGR
ENSFCAG00000013378	TTTCCTACTGCCCTGGAATG	CGTACACGAAGATGAGAAGAGAG	ATP1A1
ENSFCAG00000013165	CTTTGACCGTGTGCCTAGTT	GAGGTTGCTCACCTGGTTTAT	PAPSS2
ENSFCAG00000027930	CATCATCTGCTCCGTGATCTT	TGTCATTGATGAGGCGGATAAT	CYP2F1
ENSFCAG00000005165	CCAGAGGGAAGGTTTAGGTATG	CCCTGTCCTCATTTCCTTTCT	PLB1
ENSFCAG00000003788	TCACCCACAGCTGCAATAC	GTTCATCTCCTTCACCACACTC	DPEP1
ENSFCAG00000036330	GTTCACCATCGGCAAAGAATG	AGGGAAGTCTGCCACTATCT	FABP6
CircRNA			
novel_circ_0008741	TGGATGAGCCAATTTCCAGTCA	TGGATGAGCCAATTTCCAGTCA	
novel_circ_0010073	TTCTTGGAGGTGTGGGGTGT	TTGGATGCTCACGTCTTCTGA	
novel_circ_0009117	TCTGCATTGAACTGTTTTGTCTGT	AGCCTCATCATCACTTGCATCT	
novel_circ_0017780	TGCCGAGCAATTGTGATGAG	TAGTGAGGCAGTCCAGTTGC	
novel_circ_0002377	CTACATGGGAGGTGCACGAG	CTACATGGGAGGTGCACGAG	
novel_circ_0006332	TCACAATGGATACAGAGTCGAGTG	TAATCTGTACCTCACCTTCTTTGC	
novel_circ_0005080	AGCTGGAGACAAGGAAGTTGT	CTGCATGGCTGTACGGTCTAC	
novel_circ_0019348	CGCTACACCCTCAAGACCG	CACGGGCACAATCGTCATGG	
novel_circ_0004720	AATTCCCAAGCCAGAGCCTC	CAAGCACTGCCACTCCTCCT	
AB051104.1	GACCACCTTCAACTCCATCAT	GATCTCCTTCTGCATCCTGTC	*Felis catus* β-actin

qRT-PCR: Quantitative reverse transcription PCR; lncRNA: Long non-coding RNA; CircRNA: Circular RNA; mRNA: Messenger RNA

## Results

### *T. gondii* infection in the feline small intestinal epithelia

The PCR results revealed that the samples of the small intestinal epithelia of the infected groups were positive for *T. gondii* B1 at 6, 10, and 14 DPI and at SI, and there were no positive results for the B1 gene in the control group (Additional file 1a). Histopathological analyses revealed a clear difference between the control and infection groups (Additional file 1b). Histopathological analyses of the small intestines of cats at 6 DPI revealed infiltration of inflammatory cells into the intestinal epithelial muscle layer and submucosa (pathological section information at other DPI is shown in Additional file 1b). These results demonstrated that *T. gondii* infection was successfully established in the feline small intestinal epithelia.

### RNA sequencing and identification of ncRNAs and mRNAs in feline small intestinal epithelia

**Fig. 1:**
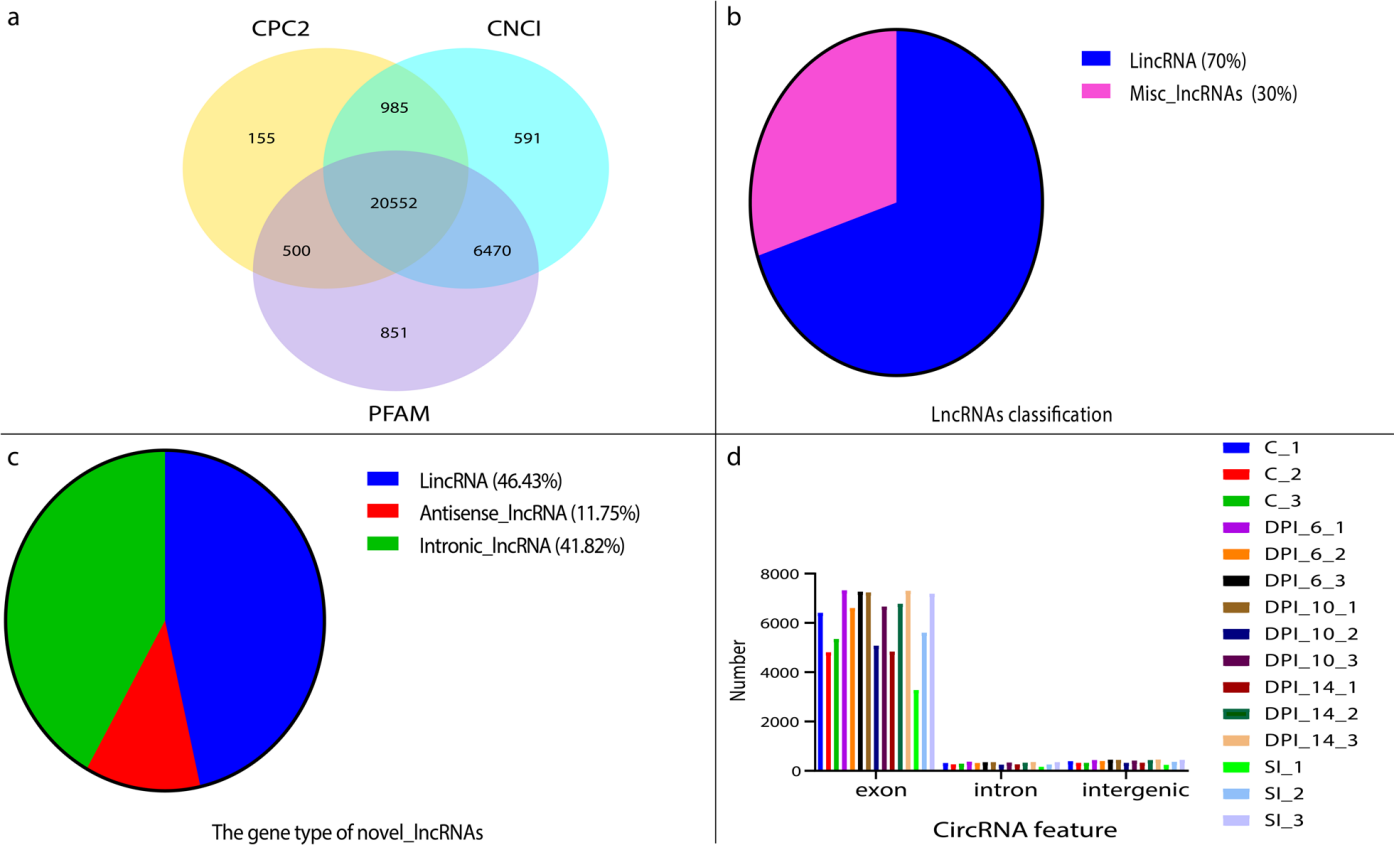
Results of classification of lncRNAs and circRNAs. Venn diagram showing the results of coding potential screening (**a**). Distribution map of different types of novel lncRNAs (**b**). The gene type of novel lncRNAs (**c**). Feature of circRNAs (**d**). Abbreviations CNCI: coding-non-coding-index, CPC2: coding potential calculator algorithm to version 2, PFAM: a widely used database of protein families (http://pfam.sanger.ac.uk/)

After RNA extraction and detection, library construction and detection, sequencing, the sequenced reads were obtained. In quality trimming, reads containing adaptor sequences, low-quality reads, and bacterial sequencing reads were filtered, and an average of 138,883,320 clean reads were obtained for each sample. Sequencing quality data is provided in Additional file 2. An average of 87.9% (range: 74.8–94.5%) of the reads were subsequently mapped to the feline reference genome using HISAT2 v2.0.4. We identified 64,748 transcripts, 207 annotated lncRNAs (lncRNA), 20,552 novel lncRNAs, and 19,409 mRNAs (Fig. 1a). The annotated lncRNAs (270) comprised of long intergenic non-coding RNAs (lincRNAs) (189, 70%) and misc_lncRNAs (81, 30%) (Fig. 1b). Similarly, the gene types of the novel lncRNAs (20,552) included lincRNAs (9544; 46.4%), antisense lncRNAs (2414; 11.8%), and intronic lncRNAs (8594; 41.8%) (Fig. 1c). In total, 3342 novel circRNAs were identified, including 2985 exons, 210 intergenic sequences, and 147 introns (Fig. 1d). In total, 2080 miRNAs were detected, of which 2167 were up-regulated and 1811 were down-regulated. The miRNAs and their corresponding target genes are listed in Additional file 3. The RNAs and their features are provided in Additional file 4.

### DE mRNAs and ncRNAs, and validation of DE RNA data

**Fig. 2:**
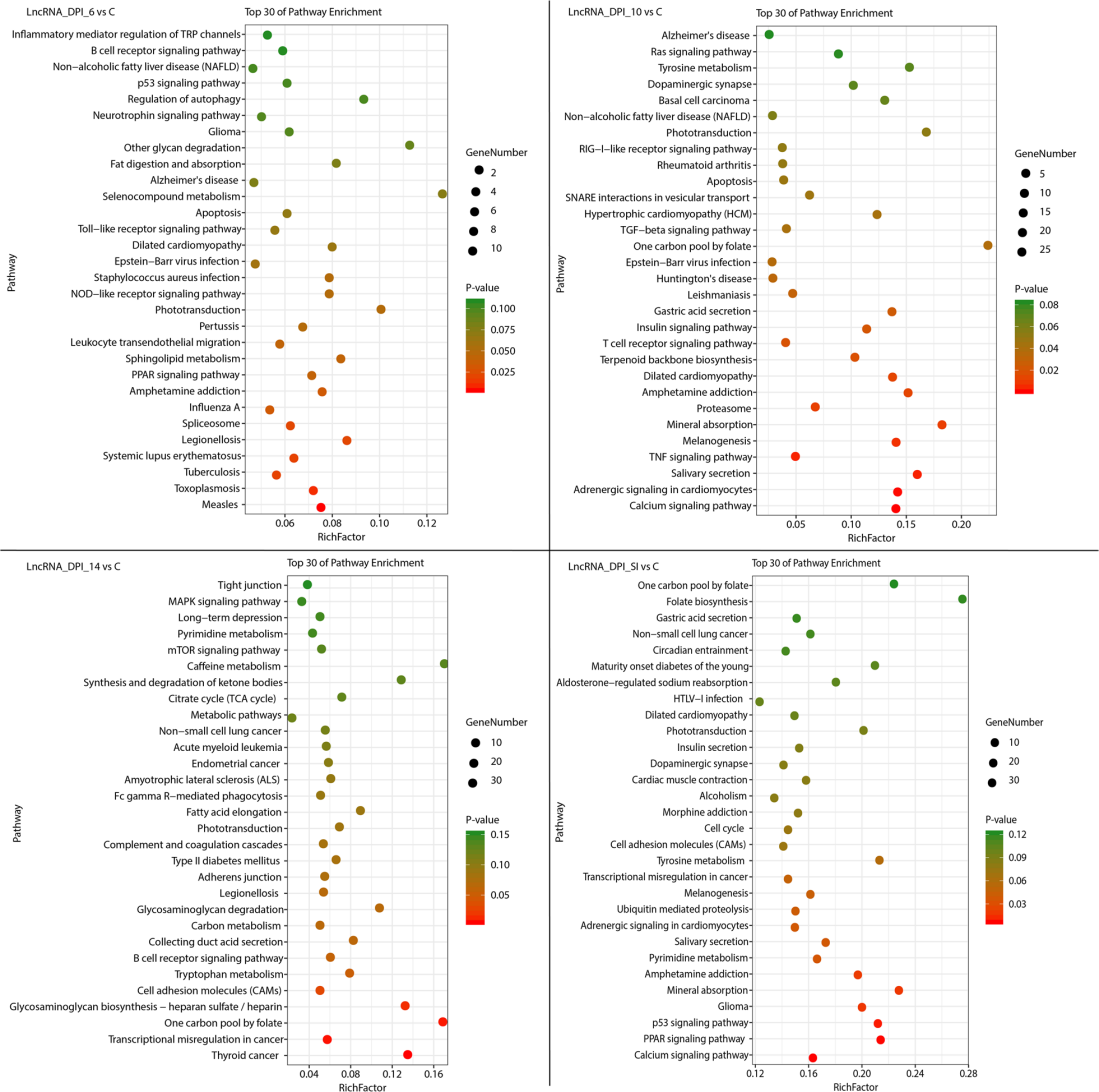
Scatterplot of the 30 most enriched KEGG pathways of the differentially expressed (DE) lncRNAs. The pathways enriched at 6, 10, and 14 days post infection (DPI) and at SI (secondary infection, 30 DPI). The y-axis represents the distinct KEGG pathways, while the x-axis represents the rich factor. The rich factor refers to the ratio of DE lncRNAs annotated in the pathway to the total number of genes annotated in the pathway. The higher the rich factor, the greater the degree of pathway enrichment. The size of the dots corresponds to the number of DE lncRNAs, with larger dots denoting a larger number of DE lncRNAs. The colours of the dots represent the *p*-values of enrichment

**Fig. 3:**
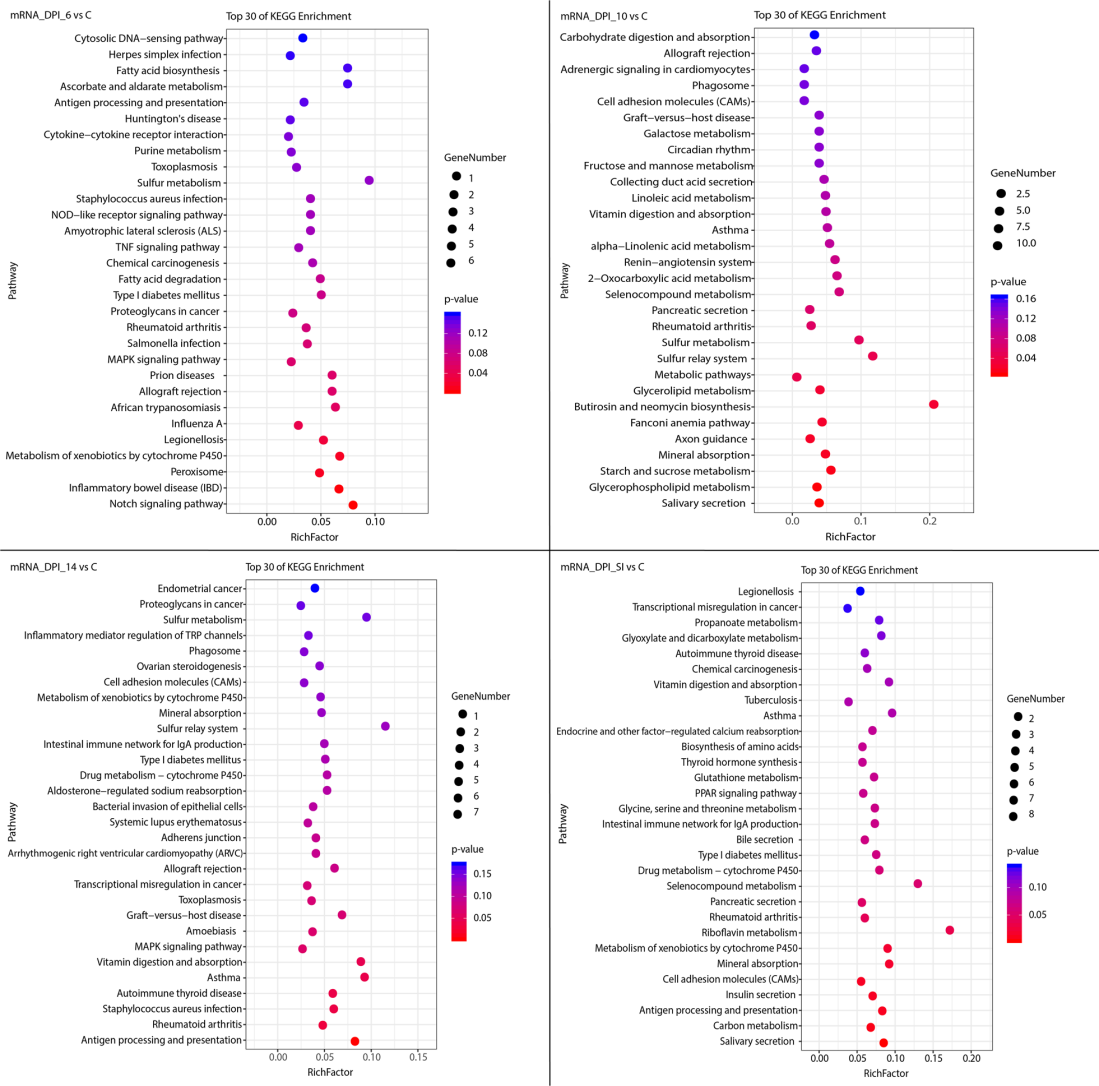
Scatterplot of the 30 most enriched KEGG pathways of the differentially expressed (DE) mRNAs. The 30 most enriched KEGG pathways at 6, 10, and 14 days post infection (DPI) and at SI (secondary infection, 30 DPI). The y-axis represents the distinct KEGG pathways, and the x-axis represents rich factor. The rich factor refers to the ratio of DE mRNAs annotated in the pathway to the total number of genes annotated in the pathway. The higher the rich factor, the greater the degree of pathway enrichment. The size of the dots corresponds to the number of DE mRNAs, with larger dots denoting a larger number of DE mRNAs. The colours of the dots represent the *P*-values (*P* < 0.05) of enrichment

In this study, 543 DE mRNAs, 358 DE lncRNAs and 407 DE circRNAs were detected in the small intestinal epithelia of infected cats. In our study, 284 lncRNAs were associated with 2441 mRNAs (Additional file 5). KEGG analyses tools was used for analyzing the biological features of the DE lncRNAs based on the dysregulated target genes identified in this study. The two most enriched KEGG pathways of the DE lncRNAs were toxoplasmosis and legionellosis at 6 DPI, calcium signaling pathway and adrenergic signaling in cardiomyocytes at 10 DPI, one carbon pool by folate and thyroid cancer at 14 DPI, and calcium and PPAR signaling pathways at SI (Fig. 2). The two most enriched pathways of DE mRNAs were the notch signaling pathway and inflammatory bowel disease (IBD) at 6 DPI, salivary secretion and glycerophospholipid metabolism at 10 DPI, antigen processing and presentation and rheumatoid arthritis at 14 DPI, and salivary secretion and carbon metabolism at SI (Fig. 3).

**Fig. 4:**
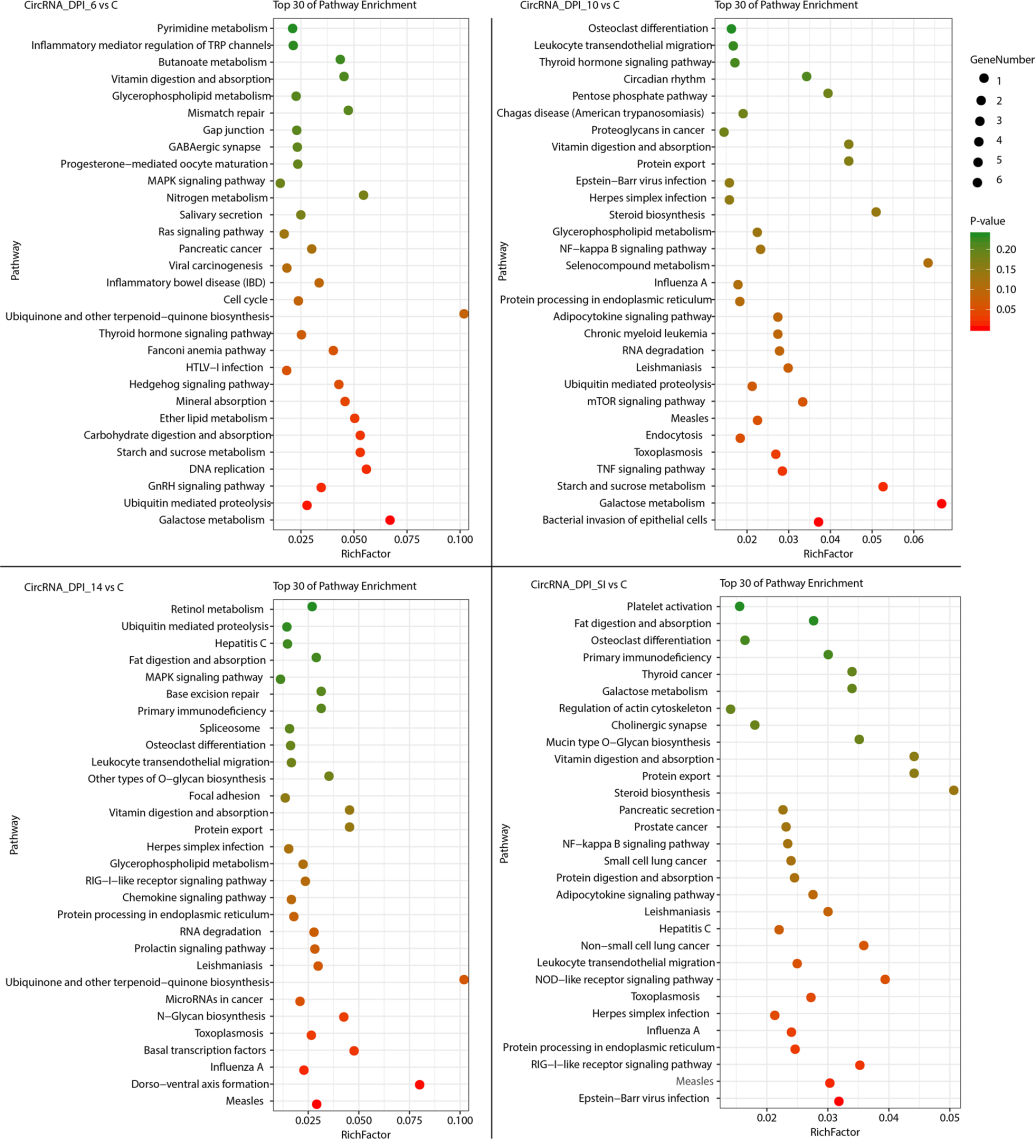
Scatterplot of the 30 most enriched KEGG pathways of the differentially expressed (DE) circRNAs. The 30 most enriched KEGG pathways at 6, 10, and 14 days post infection (DPI) and at SI (secondary infection, 30 DPI). The y-axis represents the distinct KEGG pathways, while the x-axis represents the rich factor. The rich factor refers to the ratio of DE circRNAs annotated in the pathway to the total number of genes annotated in the pathway. The higher the rich factor, the greater the degree of pathway enrichment. The size of dots represents the number of DE circRNAs, with larger dots denoting a large number of DE circRNAs. The colours of the dots represent the *p*-values of enrichment

CircRNAs inhibit miRNA function by binding to miRNAs. Therefore, analysis of the miRNA binding sites of circRNAs can aid in identifying their functions. The binding sites of 402 circRNAs to 2,082 miRNAs were predicted in this study (Additional file 3). The two significantly enriched KEGG pathways for circRNAs were galactose metabolism and ubiquitin mediated proteolysis at 6 DPI, starch and sucrose metabolism and galactose metabolism at 10 DPI, dorso–ventral axis formation and measles at 14 DPI, and Epstein-Barr virus infection and measles at SI (Fig. 4).

**Fig. 5:**
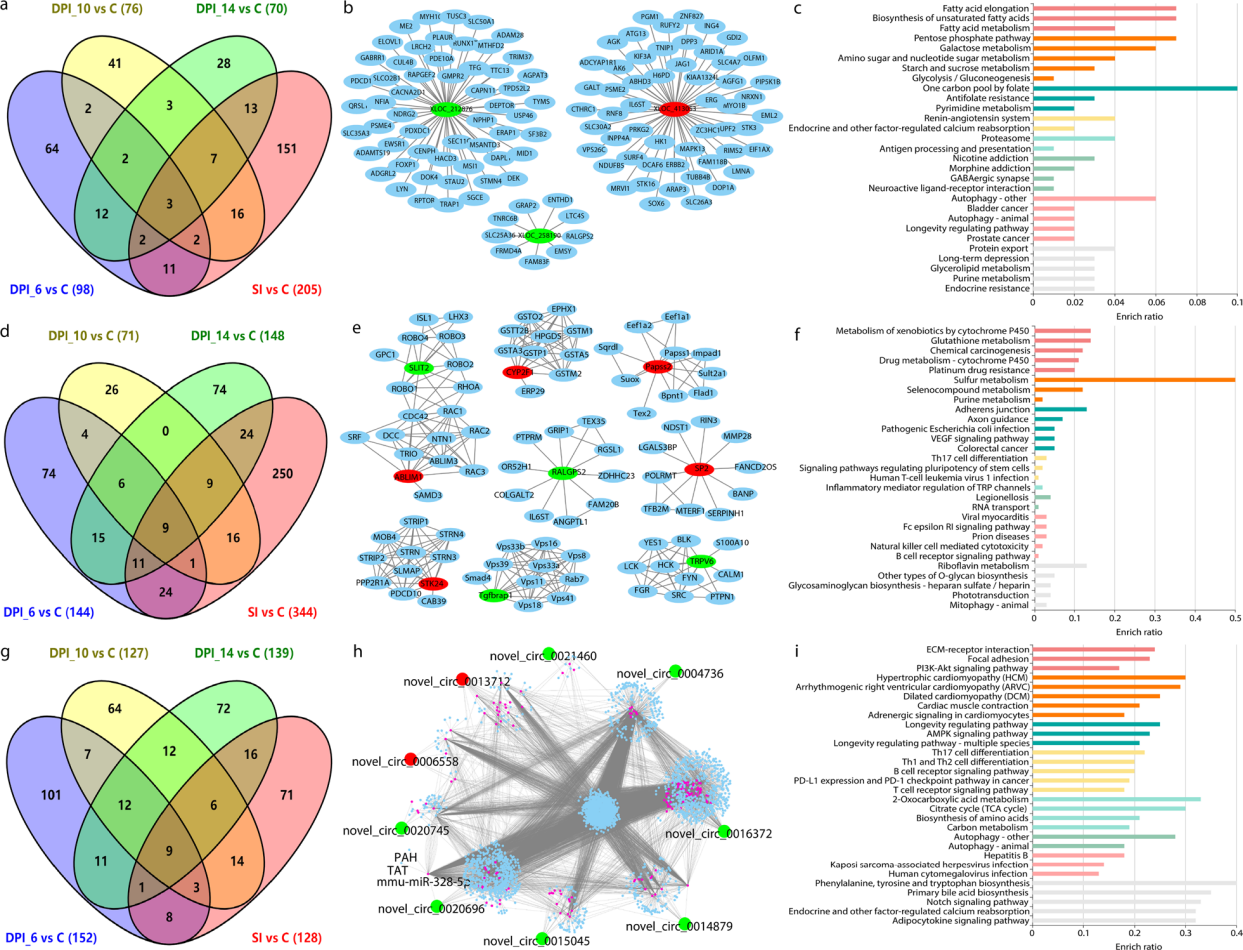
The differentially expressed lcnRNAs, mRNAs and circRNAs. Venn plot of DE lncRNAs (**a**). Target genes of common DE lncRNAs (**b**). Enrichment analysis of the target genes of common DE lncRNAs (**c**). Venn plot of DE mRNAs (**d**). Interacting proteins of common DE mRNAs (**e**). Enrichment analysis of the interacting protein of common DE mRNAs (**f**). Venn plot of DE circRNAs (**g**). Targeted gene networks of common DE circRNAs (**h**). Enrichment analysis of the target genes of common DE circRNAs (**i**). Red nodes present upregulated RNAs, Green nodes present downregulated RNAs

Of these differential RNA, 9 mRNAs (upregulated PAPSS2, CYP2F1, SP2, ABLIM1, STK24, and downregulated RALGPS2, TRPV6, TGFBRAP1, SLIT2), 9 circRNAs (upregulated novel_circ_0006558, novel_circ_0013712, and downregulated novel_circ_0004736, novel_circ_0014879, novel_circ_0015045, novel_circ_0016372, novel_circ_0020696, novel_circ_0020745, novel_circ_0021460), and 3 lncRNAs (upregulated XLOC_413053, and downregulated XLOC_212876 and XLOC_258190) were significantly altered at all four time points, namely, at 6, 10, 14 DPI and at SI (Fig. 5a, d, g). As shown in Fig. 5b, XLOC_212876 regulates 55 feline genes, XLOC_258190 regulates 6 feline genes, and XLOC_413053 regulates 54 feline genes. The functional enrichment analysis of common lncRNA-targeted genes is depicted in Fig. 5c. In this study, we found 9 commonly altered genes in the infected feline small intestine at the four time points, and these genes showed the same altered direction, such as upregulated PAPSS2, CYP2F1, SP2, ABLIM1, and STK24; and downregulated RALGPS2, TRPV6, TGFBRAP1, and SLIT2 (Fig. 5d). To determine the roles of these commonly altered genes during *T. gondii* infection in cats, we constructed protein interaction networks for these genes (Fig. 5e). As shown in Fig. 5f, the common DEGs and their interacting proteins, such as sulfur and glutathione, were mainly enriched in material metabolism. Nine common novel circRNAs (upregulated novel_circ_0006558, novel_circ_0013712, and downregulated novel_circ_0004736, novel_circ_0014879, novel_circ_0015045, novel_circ_0016372, novel_circ_0020696, novel_circ_0020745, and novel_circ_0021460) were identified at the four time points (Fig. 5g). The 9 common circRNAs had a wide range of targeted miRNAs (161 miRNAs) which regulate 2527 protein coding RNAs (Fig. 5h). The 2527 protein coding RNAs were significantly enriched in a wide range of pathways, such as the metabolic pathways and immune pathways (Fig. 5i). Volcano plots of ncRNAs and mRNAs identified in this study are depicted in Additional files 6 to 8. The reliability of the RNA sequencing results was confirmed by validating the changes in the expression of ncRNAs and mRNAs using qRT-PCR. As depicted in Additional file 9, the changes in expression (upregulation and downregulation) revealed by qRT-PCR corroborated those of RNA-seq, indicating the reliability of the transcriptomic data. The target genes of the DE lncRNAs were predicted by analysing the co-location and co-expression of lncRNAs and mRNAs.

### CeRNA regulatory network and weighted gene co-expression network analysis (WGCNA)

**Fig. 6:**
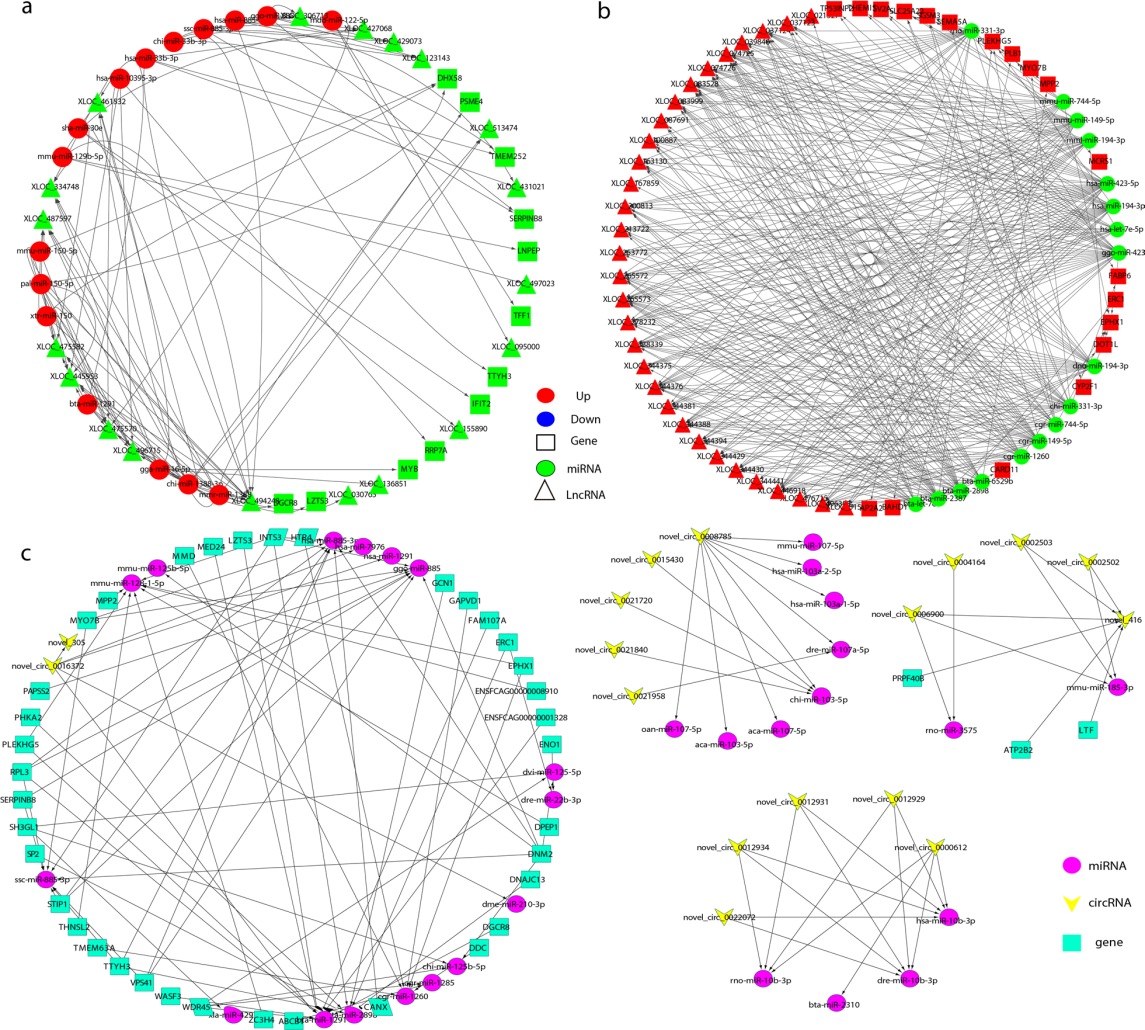
The differentially expressed (DE) lncRNA-miRNA-ceRNA regulatory network (**a**, **b**). Squares represent genes, circles represent miRNAs, and triangles represent lncRNAs. Red represents upregulation and green represents downregulation. The ceRNA regulation network of the circRNA–miRNA gene pairs (**c**), with miRNAs serving as decoys (round, pink), circRNAs as mediators (arrow-shaped, yellow), and mRNAs as target (square, dark green)

A ceRNA regulatory network analysis was performed to identify the molecular functions of ncRNAs that regulate the interplay between *T. gondii* and the small intestinal epithelia of cats. A DE lncRNA-miRNA-ceRNA regulatory network, including up-regulated and down-regulated miRNAs, lncRNAs, and target genes (Fig. 6a and b), was constructed in this study. Analyses of the DE lncRNA-miRNA-ceRNA regulatory network revealed interplay among DE ncRNAs. For instance, we observed that 9 down-regulated lncRNAs (XLOC_334748, XLOC_445953, XLOC_461532, XLOC_475570, XLOC_475582, XLOC_487597, XLOC_494248, XLOC_496715, and XLOC_513474) could competitively bind to the up-regulated chi-miR-1388-3p miRNA that targets the *PSME4* gene; while 23 up-regulated lncRNAs (XLOC_037124, XLOC_060768, XLOC_074725, XLOC_074726, XLOC_083528, XLOC_083999, XLOC_087691, XLOC_100887, XLOC_148431, XLOC_163130, XLOC_200813, XLOC_253772, XLOC_253776, XLOC_262034, XLOC_265572, XLOC_265573, XLOC_338339, XLOC_344376, XLOC_344394, XLOC_344398, XLOC_365395, XLOC_490204, and XLOC_496355) competed with 3 up-regulated mRNAs (CARD11, DOT1L, and PLEKHG5) for binding to the down-regulated bta-miR-2387 miRNA.

CircRNA-gene pairs with the same binding sites as miRNAs were analysed based on the ceRNA network theory. The ceRNA regulatory network was generated by constructing part of the circRNA–miRNA gene pairs, with miRNAs serving as decoys, circRNAs as mediators, and mRNAs as targets. As depicted in Fig. 6c, a single miRNA was directly or indirectly regulated by multiple genes and sponged by circRNAs. Novel_circ_0016372 and 9 genes (*DGCR8, ENSFCAG00000001328, FAM107A, LZTS3, MED24, PLEKHG5, RPL3, WASF3,* and *WDR45*) directly regulated bta-miR-1291 miRNA, whereas 3 miRNAs (dme-miR-210-3p, hsa-miR-1291, and hsa-miR-7976) and novel_305 indirectly regulated bta-miR-1291 through novel_circ_0016372. We observed that a single circRNA could regulate more than one miRNA. For instance, novel_circ_0008785 could regulate 8 miRNAs (chi-miR-103-5p, dre-miR-107a-5p, aca-miR-103-5p, hsa-miR-103a-1-5p, hsa-miR-103a-2-5p, aca-miR-107-5p, mmu-miR-107-5p, and oan-miR-107-5p). The details of the ceRNA network of circRNA-gene-miRNA are provided in Fig. 6c. Additional file 10 depicts the regulatory relationships between ncRNAs and mRNAs during *T. gondii* infection of cat small intestinal epithelia.

**Fig. 7:**
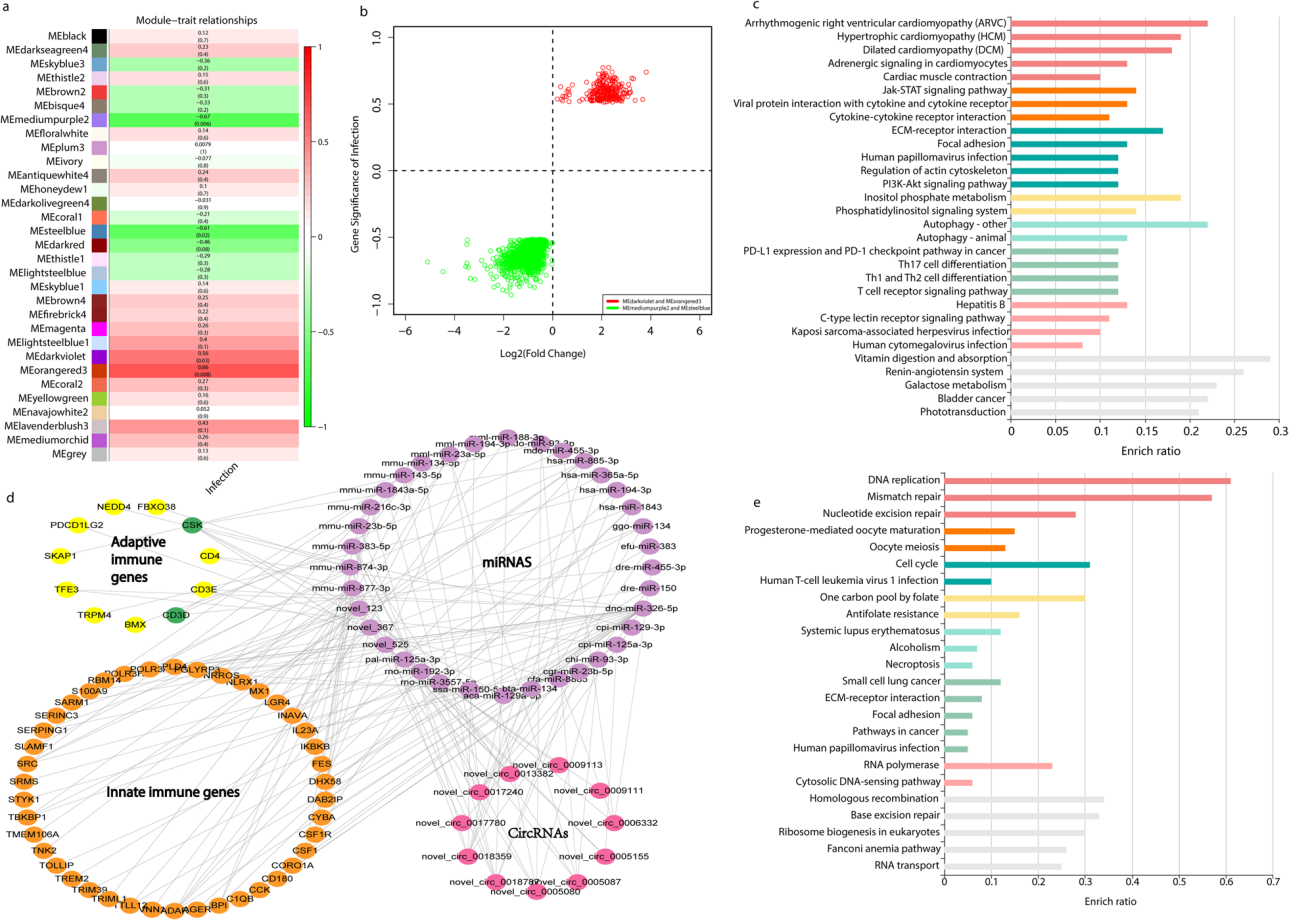
Co-expression analysis of the RNA transcripts detected in this study. CircRNA modules and relationships with *T. gondii* infection (**a**). Relationship between gene significance of infection and gene expression Log2 (fold change) of infected samples/non-infected samples (**b**). The significantly enriched pathways of target genes of circRNAs (circRNAs in MEdarkviolet or MEorangered3 modules) that were positively associated with *T. gondii* infection (**c**). Regulation networks of the positively associated circRNAs that were involved in immune response (**d**). The significantly enriched pathways of protein coding RNAs (The protein coding RNAs in MEmediumpurple2 and MEsteelblue modules) that were negatively associated with *T. gondii* infection (**e**)

To identify the genes that were significantly associated with *T. gondii* infection, we performed WGCNA for systematically identifying the genes that were significantly associated with specific treatments or infection process. As shown in Fig. 7a, the expression module MEmediumpurple2, MEsteelblue, MEdarkviolet and MEorangered3 were significantly associated with *T. gondii* infection. MEmediumpurple2 and MEsteelblue showed negative associations, whereas MEdarkviolet and MEorangered3 showed positive associations. In the MEdarkviolet and MEorangered3 modules, 260 genes were positively associated with *T. gondii*, including 1 lncRNA, 242 circRNAs, and 16 protein-coding RNAs. In the MEmediumpurple2 and MEsteelblue modules, 44 lncRNAs and 1352 protein-coding RNAs were negatively associated (Fig. 7b and Additional file 11). In this study, we found that 188 miRNAs could bind to 242 positively associated circRNAs, and 1,726 genes could be regulated by 188 miRNAs. KEGG enrichment of the 1726 genes is shown in Fig. 7c. As shown in Fig. 7c, 12 pathways were involved in immune or infection responses, including the Jak-STAT signaling pathway, viral protein interaction with cytokine and cytokine receptor, cytokine-cytokine receptor interaction, human papillomavirus infection, Th17 cell differentiation, Th1 and Th2 cell differentiation, T cell receptor signaling pathways, hepatitis B, C-type lectin receptor signaling pathway, Kaposi sarcoma-associated herpesvirus infection, human cytomegalovirus infection, and galactose metabolism. In this study, we found that 11 circRNAs positively associated *T. gondii* infection regulated 55 immune response genes by binding to 36 miRNAs (Fig. 7d). The KEGG enrichment of protein-coding RNAs that were negatively associated with *T. gondii* infection is shown in Fig. 7e. As shown in Fig. 7e, 8 pathways were associated with nucleic acids and 2 pathways were associated with oocyte development.

## Discussion

*Toxoplasma gondii* is a worldwide spreading pathogen that causes stillbirth, brain damages, and psychiatric disorders in infected patients. Generally, toxoplasmosis is caused by *T. gondii* infection resulting from the consumption of undercooked or raw meat containing tissue cysts or the ingestion of vegetables, fruits, soil, and water contaminated by sporulated oocysts [51]. Toxoplasmosis can be transmitted from an infected pregnant woman to her fetus [52]. This can result in severe health issues, including neurological and visual impairments, intellectual disabilities, and even stillbirth or miscarriage. *T. gondii* can also be transmitted from infected animals to humans, especially through handling cat feces or direct contact with infected soil [53]. Proper hygiene practices and preventive measures are essential to minimize the risk of infection. All warm-blooded animals and humans can be intermediate hosts of *T. gondii* [54]; however, felids are the only definitive hosts of *T. gondii*, and sexual reproduction of *T. gondii* occurs only in the small intestinal epithelia of cats. Numerous studies have aimed to elucidate the mechanism of sexual reproduction in *T. gondii*, and mRNA profiles of the small intestine of cats following *T. gondii* infection have been reported [9, 34]. However, the mechanisms underlying the sexual reproduction of *T. gondii* remain unclear. RNAs only account for a small fraction of the RNA profile, and ncRNAs, including lncRNAs, circRNAs and miRNAs, play essential roles in host–pathogen interactions [55–58]. The dissection of the RNA profile of the feline small intestine after *T. gondii* infection is essential for the development of novel methods to control and cure toxoplasmosis. However, information regarding the role of ncRNAs in the small intestine of cats during primary or secondary infection with *T. gondii* is limited. In this study, we analysed the profiles of ncRNAs and coding RNAs in the small intestinal epithelia of cats infected with *T. gondii*. Histological analysis and PCR results showed that the cat small intestine model infected with *T. gondii* was successfully established at different intervals (Additional file 1).

In this study, a total of 64,748 transcripts, 207 annotated lncRNAs, 20,552 novel lncRNAs, and 19,409 mRNAs were identified as high-quality transcripts in the infected feline small intestines (Fig. 1a, b, and Additional file 2). Novel lncRNAs (20,552) were classified as lincRNAs (9544, 46.4%), antisense lncRNAs (2414, 11.8%), and intronic lncRNAs (8594, 41.8%) (Fig. 1c). In total, 3342 novel circRNAs were identified (Fig. 1d). Feline miRNAs and their corresponding target circRNAs were predicted in this study, and 6186 interacting relationships were identified (Additional file 3). The RNAs and their features are provided in Additional file 4.

LncRNAs are important regulators of infection and disease development, and their potential functions are associated with the functions of their target genes. However, the roles of lncRNAs in the interplay between *T. gondii* and its definitive host remain unclear. The KEGG enrichment map of the DE lncRNAs is depicted in Fig. 2, showing that measles and toxoplasmosis were the two most significantly enriched KEGG pathways at 6 DPI, which are often detected and studied along with toxoplasmosis [59–62]. At 10 DPI, calcium signaling pathway and adrenergic signaling in cardiomyocytes pathway were significantly enriched. The calcium signaling pathway regulating *T. gondii* infectivity reveals interrelationships within the second messenger pathways of *T. gondii* in different intracellular compartments and at different time points during infection [63]. Adrenergic signaling in cardiomyocytes pathway was also enriched at 10 DPI; however, the role of this pathways in *T. gondii* infection in cats remains unclear. A previous study demonstrated that *T. gondii* can alter the p53 cancer signaling pathway, which plays an important role in the development of human cancers [64], and significantly contributes to the tumour suppressor function of p53, which regulates the transcription of genes associated with diverse cellular functions [65]. The majority of DE lncRNAs that regulate the p53 signaling pathway in the secondary infection group were significantly downregulated, suggesting that *T. gondii* infection can alter the p53 signaling pathway by altering the expression of lncRNAs [66]. In this study, the PPAR signaling pathway was enriched at both 6 DPI and SI. Previous transcriptomic and proteomic studies demonstrated that this pathway is downregulated in the kidneys of mice infected with *T. gondii* [67, 68]. However, the role of lncRNAs in triggering PPAR signaling pathway alteration during *T. gondii* infection (especially during the second infection) in cats remains limited. The results of this study demonstrate, for the first time, that the feline PPAR pathway can be affected by alterations in lncRNAs.

The KEGG enrichment map of the DE mRNAs is depicted in Fig. 3, showing that the notch signaling pathway was significantly altered at 6 DPI. The notch signaling pathway is the main factor driving the production of IL-10 by Th1 cells [69, 70], which are the primary source of IL-10 during *T. gondii* infection in mice [71]. Alterations in the notch signaling pathway may contribute to the regulation of cellular immune responses in the feline small intestine during *T. gondii* infection. In addition to the notch signaling pathway, several immune pathways were also altered during *T. gondii* infection in cats, such as antigen processing and presentation, and MAPK signaling pathway. Interestingly, PPAR signaling pathway was not enriched in DE mRNA in this study, but was enriched in mouse and pig models [67, 72]. This could be the result of the different biological characteristics of the infected hosts. Although the PPAR signaling pathway was not enriched by DE mRNA in cats, it was enriched by DE feline lncRNA-targeted genes. Previous studies have confirmed that lncRNAs play important roles in post-transcriptional translation and that gene expression at the protein level differs from that at the mRNA level [73]. Although the PPAR signaling pathway was not enriched by DE mRNA, it is likely that the PPAR signaling pathway could be altered during the post-transcriptional translation process, and post-transcriptional translation regulation could be an important difference between definitive and intermediate hosts.

CircRNAs act as miRNA sponges, adsorbs miRNAs, and play critical roles in biological regulation [74]. To date, there has been no circRNA research on the feline small intestine after *T. gondii* infection. In this study, 3342 novel feline circRNAs were identified, 402 of which were predicted to bind to 2082 miRNAs. Following *T. gondii* infection, 407 DE circRNAs were identified in the feline small intestine. The results of the KEGG enrichment analysis of DE circRNAs are depicted in Fig. 4. Our circRNA analysis showed that circRNAs play roles in regulating the feline immune response against *T. gondii* infection, and that several immune pathways (such as TNF signaling pathway, protein processing in endoplasmic reticulum, and galactose metabolism pathway) are regulated by DE circRNAs. The recognition of galactose by the TgMIC4 protein of *T. gondii* may impair host protection by regulating galectin-mediated activation of the host immune system [75]. The TgMIC4 protein has a high specificity for galactose owing to the high binding specificity of the galactose-end group [76]. In this study, the galactose metabolite pathway was significantly enriched at both 6 DPI (start of oocysts shedding) and 10 DPI (the peak shedding period of oocysts) using the genes regulated by DE circRNAs. Thus, the results of the circRNA analysis in this study indicated that circRNAs could be crucial regulators of the immune response in cat post-infection with *T. gondii*.

Altered genes play important roles in the interplay between a pathogen and its host. In this study, we observed that 3 common DE lncRNAs (Fig. 5a, b) that regulated 118 genes were significantly altered at all four time points. Functional enrichment analysis showed that the targets of the common lncRNAs were mainly involved in the biosynthesis of unsaturated fatty acids (Fig. 5c). A previous study showed that linoleic acid, an unsaturated fatty acid, plays a key role in sexual reproduction of *T. gondii* in cats [77]. Therefore, it is likely that *T. gondii* can use these common feline DE lncRNAs to promote transmission in cats. In this study, we found 9 common DE mRNAs in the infected feline small intestine at four time points, and these genes showed the same altered direction, such as upregulated PAPSS2, CYP2F1, SP2, ABLIM1, STK24, and downregulated RALGPS2, TRPV6, TGFBRAP1, SLIT2 (Fig. 5d). To determine the roles of these commonly altered genes during *T. gondii* infection in cats, we constructed protein interaction networks for these genes (Fig. 5e). As shown in Fig. 5f, the common DEGs and their interacting proteins, such as sulfur and glutathione, were mainly enriched in material metabolism. The results of the present study are consistent with the finding that a statistically significant difference in glutathione levels was observed in hosts infected with *T. gondii* [78]. We also identified 9 common DE circRNAs (upregulated novel_circ_0006558, novel_circ_0013712, and downregulated novel_circ_0004736, novel_circ_0014879, novel_circ_0015045, novel_circ_0016372, novel_circ_0020696, novel_circ_0020745, novel_circ_0021460) in the infected feline small intestine (Fig. 5g). A total of 161 miRNAs were predicted to be regulated by the 9 circRNAs, and 2527 protein coding RNAs were indirectly regulated by the 9 common DE circRNAs (Fig. 5h). The 2527 protein coding RNAs were significantly enriched in a wide range of pathways, including metabolic pathways, immune pathways and phenylalanine, tyrosine and tryptophan biosynthesis pathway (Fig. 5i). Phenylalanine, tyrosine and tryptophan biosynthesis pathway are essential for the infection of *T. gondii* in cats. A previous study showed that the knock-out of *AAH1* and *AAH2* in *T. gondii* can reduce *T. gondii* infection in cats, lower oocyst yields, and decrease sporulation rates [79]. *AAH1* and *AAH2* of *T. gondii* can catalyse the conversion of phenylalanine to tyrosine, and then convert tyrosine to 3,4 dihydroxyphenylalanine (L-DOPA) [80], which is critical for the survival of *T. gondii* [81]. In this study, we found that by targeting mmu-miR-328-5p, novel_circ_0020696 could regulate phenylalanine hydroxylase (PAH) and tyrosine aminotransferase (TAT) in cats, which participate in the phenylalanine, tyrosine and tryptophan biosynthesis pathway. Therefore, the novel_circ_0020696 may be an important non-coding RNA in the interplay between cats and *T. gondii*.

Analysis of ncRNAs and their roles in the ceRNA network may provide novel insights into the life cycle of *T. gondii*. Analysis of the interaction network of ncRNAs and mRNAs revealed that several ncRNA-miRNA-gene pairs have complex interrelationships. Target mRNAs of DE lncRNAs and DE circRNAs have been found to play critical roles in human diseases associated with *T. gondii* infection, including Parkinson’s disease and SLE [82, 83]. Interestingly, we observed that both bta-miR-2898 and mmu-miR-744-5p were targeted by XLOC_413053, while *DNAJC13* is targeted by bta-miR-2898, and *FBXW11* by mmu-miR-744-5p. The lncRNA-miRNA-gene ceRNA network is particularly important for regulating cellular biological process [84]. Interestingly, the expression of lncRNAs and mRNAs was synchronous, whereas that of miRNAs was contrary to that of the lncRNAs and mRNAs (Fig. 6a and 6b). Therefore, dissecting the ceRNA network of bta-miR-2898, mmu-miR-744-5p, *DNAJC13*, and *FBXW11* mediated via XLOC_413053 could be important for understanding the interactions between *T. gondii* and the small intestinal epithelia of cats, and help to elucidate the sexual reproduction mechanism of *T. gondii*.

Several studies confirmed that genes with similar expression patterns have similar biological functions. WGCNA is a powerful tool for analysing the gene expression associated with diseases and other traits with high accuracy [85, 86]. In this study, the mRNAs, lincRNAs and circRNAs were classified into several expression modules (Fig. 7a). As shown in Fig. 7a, the MEmediumpurple2 and MEsteelblue expression modules were negatively associated with *T. gondii* infection, whereas MEdarkviolet and MEorangered3 showed a positive association. A total of 242 circRNAs, 1 lncRNA, and 16 protein-coding RNAs were classified in the MEdarkviolet and MEorangered3 modules. In contrast, 44 lncRNAs and 1,352 protein-coding RNAs were classified in the MEmediumpurple2 and MEsteelblue modules (Fig. 7b and Additional file 11). Interestingly, the majority of the MEdarkviolet and MEorangered3 that positively associated with *T. gondii* infection were circRNAs, while the majority genes of the MEmediumpurple2 and MEsteelblue modules that were negatively associated with *T. gondii* infection were protein-coding RNAs. A previous analysis of the DE circRNAs in this study showed that they mainly participate in immune regulation. We speculated that circRNAs positively associated with infection could also play important roles in the immune response against *T. gondii* infection. Consistent with our speculation, our pathway enrichment analysis of the 1726 genes that were indirectly regulated by the circRNAs showed that 12 pathways involved in immune or infection responses were significantly enriched, including the Jak-STAT signaling pathway, cytokine-cytokine receptor interaction, galactose metabolism, and T cell receptor signaling pathway. The regulation network analysis showed that 11 circRNAs regulated 55 innate or adaptive immune response genes by binding to 36 miRNAs (Fig. 7d). The results of this study revealed, for the first time, that circRNAs in cats play important roles in regulating adaptive or innate immune responses against *T. gondii*.

We wondered why so many protein-coding RNAs were negatively associated with *T. gondii* infection. Therefore, the pathway enrichment of genes negatively associated with *T. gondii* infection was also performed. As shown in Fig. 7e, 8 pathways were associated with nucleic acids (DNA replication, mismatch repair, nucleotide excision repair, cytosolic DNA-sensing pathways, homologous recombination, base excision repair, RNA polymerase, and RNA transport), and 2 pathways (progesterone-mediated oocyte maturation, and oocyte meiosis) were associated with oocyte development. However, it remains unclear why *T. gondii* downregulated these pathways in the small intestine of felines during sexual reproduction. However, the sexual reproduction of *T. gondii* is rapid, and loss of intestinal delta-6-desaturase activity in cats is critical for the process [77]. Whether the downregulation of these pathways or genes contributes to the sexual reproduction of *T. gondii* is an important question for elucidating its sexual reproduction. *T. gondii* infects nearly one-third of the world's population. *T. gondii* has been discovered for more than one hundred years*.* However, the mechanisms underlying sexual reproduction in *T. gondii* remain unknown. Dissecting the functions of these downregulated pathways or genes in the interplay between cats and *T. gondii* could help us design a better way to control this important pathogen. Our findings provide valuable data and clues for elucidating the interplay between *T. gondii* and its definitive host during gamogenesis and secondary challenges with *T. gondii*. However, one of the limitations of this study is that before the development of oocysts, *T. gondii* has several biological types in cat small intestinal epithelia, including a-e merozoites, macrogametocytes and microgametocytes. Using single-cell RNA-seq to study the interplay between *T. gondii* and cats could provide more details on the secretory life of *T. gondii* and how cats respond to *T. gondii* infection.

## Conclusions

In this study, we describe the comprehensive landscapes of mRNAs, lncRNAs, and circRNAs in the small intestinal epithelia of cats following *T. gondii* infection. A total of 543 DE mRNAs, 358 DE lncRNAs, and 407 DE circRNAs were identified in feline small intestinal epithelia. The DE mRNAs, DE lncRNAs and DE circRNAs were significantly enriched in metabolic processes and immune responses. The ceRNA network analysis revealed that these DE ncRNAs might play important roles in the pathogenesis of *T. gondii* infection by regulating their target miRNAs and mRNAs. In addition, we found that more than one thousand protein coding genes were significantly inversely associated with *T. gondii* infection, and these genes were significantly enriched in nucleic acid-related and oocyte development pathways.

The findings of this study provide novel insights into the altered pathways and the construction of a catalogue of DE ncRNAs in the feline small intestine following *T. gondii* infection. These findings could aid further studies on the mechanisms of sexual reproduction of *T. gondii* and the interaction between *T. gondii* and its definitive cat hosts, which will facilitate the development of novel intervention strategies against *T. gondii* infection in humans and animals.

## Supplementary Information

**Additional file 1. **Verification of *T. gondii* infection in the small intestine of cats using PCR (a). DNA was extracted from the small intestinal epithelia of infected and non-infected cat at 6, 10, and 14 days post infection (DPI) and at SI (secondary infection, at 30 DPI) to detect *T. gondii* B1 gene. The order of the sample holes is: M: Trans 500 plus DNA marker, P: *T. gondii* Pru strain PCR positive control, N: negative standard product, 6 DPI (lanes 5–7), 10 DPI (lanes 9–11), 14 DPI (lanes 13–15), SI DPI (lanes 17–19), control (lanes 21–23). The results of histopathological examination between the control and infection groups (b). At DPI_6 showed the early clinical symptoms of cat's small intestinal epithelial infection. DPI_6_a1 showed that the cat's small intestinal epithelia was fractured. DPI_6_b1 showed inflammatory cell infiltration in the intestinal epithelial muscle layer and submucosa of the cat. At DPI_10 displayed a typical acute clinical symptom, with intestinal mucosal hemorrhage (DPI_10_c1), intestinal crypt atrophy and massive inflammatory cell infiltration (DPI_10_d1). DPI_14 was the result of a chronic accumulation of clinical pathological changes. Accompanied by massive bleeding of intestinal villi (DPI_14_e1, DPI_14_f1), and intestinal villi rupture, thickening of the muscular propria and muscle layers (DPI_14_f1). The location indicated by the arrow is the lesion site.

**Additional file 2. **Summary of the quality of the sequencing data in this study (lncRNAs and circRNAs). Raw reads: statistics of raw sequence data. Clean reads: sequencing data after conditional filtering. Clean bases: the number of sequenced sequences is multiplied by the length of the sequence and converted to G units. Error rate: average base sequencing error rate. Q20, Q30: calculate the percentage of bases with phred values greater than 20 and 30 in the total bases, respectively. GC content: the sum of the number of bases G and C as a percentage of the total number of bases.

**Additional file 3. **Results of circRNA and miRNA binding site analysis. Sheet 1: miRNA target gene prediction. Target gene prediction was performed on the known and novel miRNAs obtained by the analysis, and the corresponding relationship between miRNAs and target genes was obtained. Sheet 2: Analysis results of miRNA and circRNA binding sites. The miRNA and circRNA sequences were matched, and the thermal stability and sequence conservation of the double-stranded were analyzed. The first column is the miRNA ID, the second column is the circRNA ID, the third column is the total score value of all binding sites predicted by targeting relationship (the higher the value, the higher the possibility of targeting the set), the fourth column is the total energy value (the lower the energy value, the higher the energy required to disassemble the miRNA-circRNA interaction, and the more stable the binding. That is, the target-binding relationship of the energy value is more reliable), the fifth column is the target binding to all binding sites, and the highest score is in the middle (higher value indicates a higher probability of targeting ensemble); the sixth column is the lowest energy value among all binding sites for targeted binding, the seventh column is Strand, the eighth column is miRNA length, the ninth column is circRNA length, and the tenth column is the combination position. If multiple positions can be combined, the multiple positions are separated by spaces.

**Additional file 4. **Expressional data of all the RNAs and their feature information obtained in this study. Sheet 1: The detailed information of the spliced transcripts and the partial transcripts of the known lncRNAs with overlaps in the database can be divided into intergenic lncRNAs (lincRNAs for short), intronic lncRNAs, anti-sense lncRNAs, and sense lncRNAs according to their positional relationship with the coding sequences, bidirectional lncRNA and other types. Sheet 2: Details of the novel_lncRNA screened this study. Sheet 3: Detailed property information of the mRNA of their species. Sheet 4: Annotation files of all identified circRNAs (chr: chromosome number, start: start site of full-length circRNA, end: termination site of full-length circRNA, strand: positive and negative strands, full_length: full-length circRNA, spliced_length: length of circRNA after shearing). Sheet 5: miRNA differential expression analysis results (sRNA: miRNA mature body id).

**Additional file 5. **Information on lncRNAs and target mRNAs. Co_located: position-related target gene analysis, cis target gene prediction is performed based on the positional relationship between lncRNA and mRNA, and the screening range is within 100 k. Co_expressed: target gene expression correlation analysis, target gene prediction is performed based on the expression correlation between lncRNA and mRNA, and the screening condition is that the correlation coefficient is greater than 0.95.

**Additional file 6. **Volcano map of the differentially expressed (DE) lncRNAs. Red represents upregulation, blue indicates downregulation, gray indicates insignificant change. The factor of *Q*-value < 0.05 were used as the conditions for screening differential transcripts. Total transcripts: total number of transcripts.

**Additional file 7. **Volcano map of the differentially expressed (DE) mRNAs. Red represents upregulation, blue indicates downregulation, gray indicates insignificant change. The factor of *Q*-value < 0.05 were used as the conditions for screening differential transcripts. Total transcripts: total number of transcripts.

**Additional file 8. **Volcano map of the differentially expressed (DE) circRNAs. Red represents upregulation, blue indicates downregulation, gray indicates insignificant change. The factor of *P*-value < 0.05 were used as the conditions for screening differential circRNAs. Total circRNAs: total number of circRNAs.

**Additional file 9. **Verification of the RNA-seq data using qRT-PCR. qRT-PCR and RNA-Seq are represented by red, green boxes, respectively, the upper part of the x-axis represents up-regulation, and the lower part represents down-regulation. Bars represent the mean fold changes of the expression of *T. gondii* genes. Log_2_foldchange (M/C): log_2_foldchange (expression of infection group/expression of control group).

**Additional file 10. **The lncRNA-miRNA-mRNA and circRNA-miRNA-mRNA regulatory networks constructed in this study.

**Additional file 11. **The mRNA, lncRNA and circRNA of MEmediumpurple2, MEsteelblue, MEdarkviolet and MEorangered3 modules that significantly associated with *T. gondii* infection.

## Data Availability

The datasets supporting the findings of this article are included within the paper. The RNA-Seq data obtained in this study have been deposited in the National Center for Biotechnology Information (NCBI) Sequence Read Archive (SRA) database (https://www.ncbi.nlm.nih.gov/sra) under accession numbers SUB8024024 (PRJNA658394), SUB7944975 (PRJNA658397), SUB8028854 (PRJNA658398), SUB8028086 (PRJNA658399) and SUB8022553 (PRJNA658401). Mendeley Data database also uploaded our data (https://data.mendeley.com/datasets/x9xmmx7mfw/draft?a=b7ce2ac5-8bfa-4ca9-8d79-28a7145742a0).

## Abbreviations

lncRNA: Long non-coding RNA
CircRNA: Circular RNA
DE: Differentially expressed
qRT-PCR: Quantitative reverse transcription PCR
AIDS: Acquired immunodeficiency syndrome
CNS: Central nervous system
ncRNA: Non-coding RNA
miRNA: MicroRNA
UTR: Untranslated region
ceRNA: Competing endogenous RNA
ELISA: Enzyme linked immunosorbent assay
MAT: Modified agglutination test
SI: Secondary infection
DPI: Day post infection
FPKM: Fragments per kilobase of transcript per million mapped read
TPM: Transcript per million
DEG: Differential expression gene
WGCNA: Weighted gene co-expression network analysis
MF: Molecular function
CC: Cellular component
BP: Biological process
KEGG: Kyoto Encyclopedia of Genes and Genomes
