# Supplementary figures and images for: Dynamic RNA profiles in the small intestinal epithelia of cats after Toxoplasma gondii infection
Source: Infect Dis Poverty. 2023 Jul 25;12:68. doi: 10.1186/s40249-023-01121-z (PMC10367386; doi:10.1186/s40249-023-01121-z)

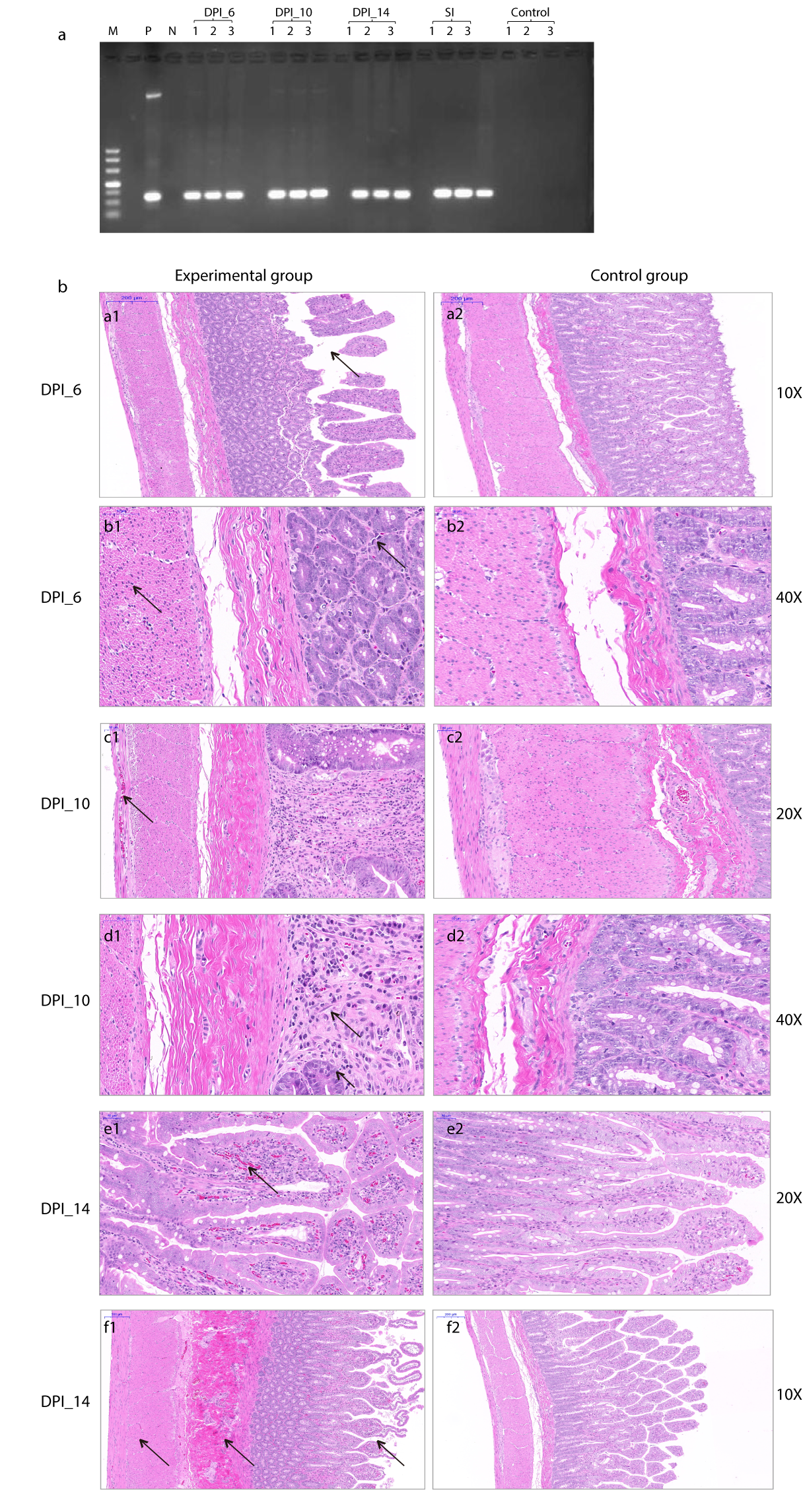

Supplement: Supplementary file 1 — Additional file 1. Verification of T. gondii infection in the small intestine of cats using PCR (a). DNA was extracted from the small intestinal epithelia of infected and non-infected cat at 6, 10, and 14 days post infection (DPI) and at SI (secondary infection, at 30 DPI) to detect T. gondii B1 gene. The order of the sample holes is: M: Trans 500 plus DNA marker, P: T. gondii Pru strain PCR positive control, N: negative standard product, 6 DPI (lanes 5–7), 10 DPI (lanes 9–11), 14 DPI (lanes 13–15), SI DPI (lanes 17–19), control (lanes 21–23). The results of histopathological examination between the control and infection groups (b). At DPI_6 showed the early clinical symptoms of cat's small intestinal epithelial infection. DPI_6_a1 showed that the cat's small intestinal epithelia was fractured. DPI_6_b1 showed inflammatory cell infiltration in the intestinal epithelial muscle layer and submucosa of the cat. At DPI_10 displayed a typical acute clinical symptom, with intestinal mucosal hemorrhage (DPI_10_c1), intestinal crypt atrophy and massive inflammatory cell infiltration (DPI_10_d1). DPI_14 was the result of a chronic accumulation of clinical pathological changes. Accompanied by massive bleeding of intestinal villi (DPI_14_e1, DPI_14_f1), and intestinal villi rupture, thickening of the muscular propria and muscle layers (DPI_14_f1). The location indicated by the arrow is the lesion site. [file 40249_2023_1121_MOESM1_ESM.tif]

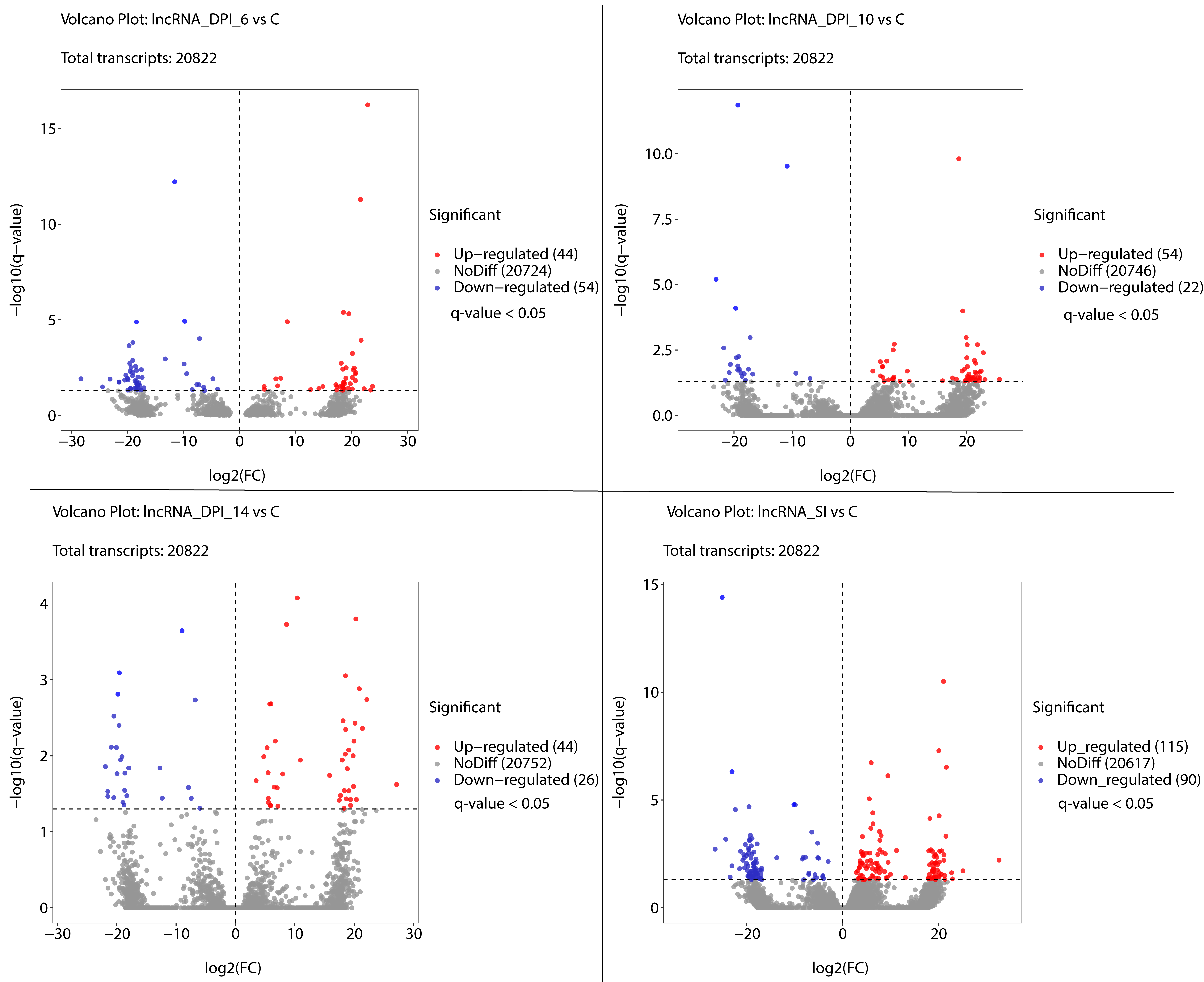

Supplement: Supplementary file 6 — Additional file 6. Volcano map of the differentially expressed (DE) lncRNAs. Red represents upregulation, blue indicates downregulation, gray indicates insignificant change. The factor of Q-value < 0.05 were used as the conditions for screening differential transcripts. Total transcripts: total number of transcripts. [file 40249_2023_1121_MOESM6_ESM.tif]

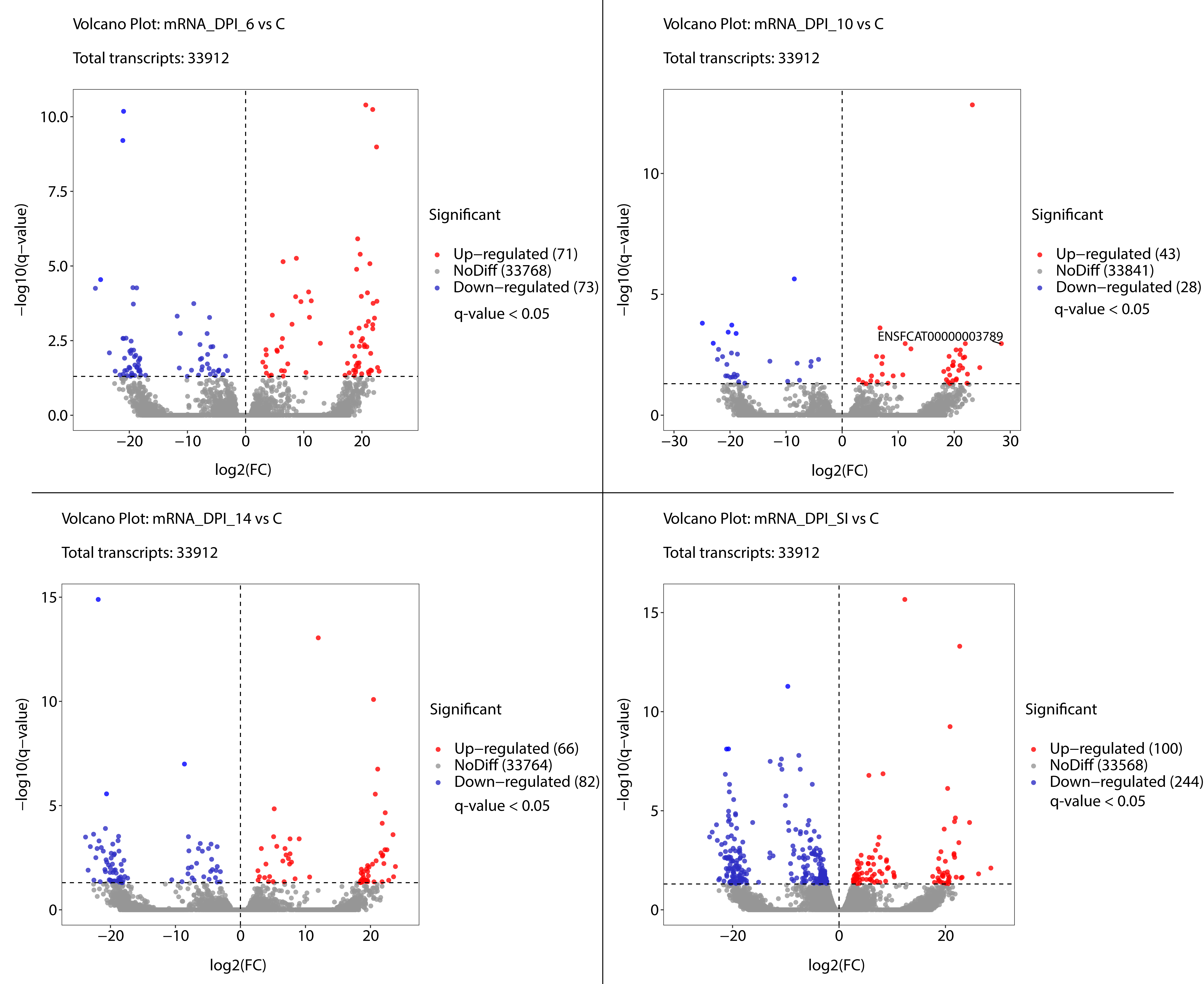

Supplement: Supplementary file 7 — Additional file 7. Volcano map of the differentially expressed (DE) mRNAs. Red represents upregulation, blue indicates downregulation, gray indicates insignificant change. The factor of Q-value < 0.05 were used as the conditions for screening differential transcripts. Total transcripts: total number of transcripts. [file 40249_2023_1121_MOESM7_ESM.tif]

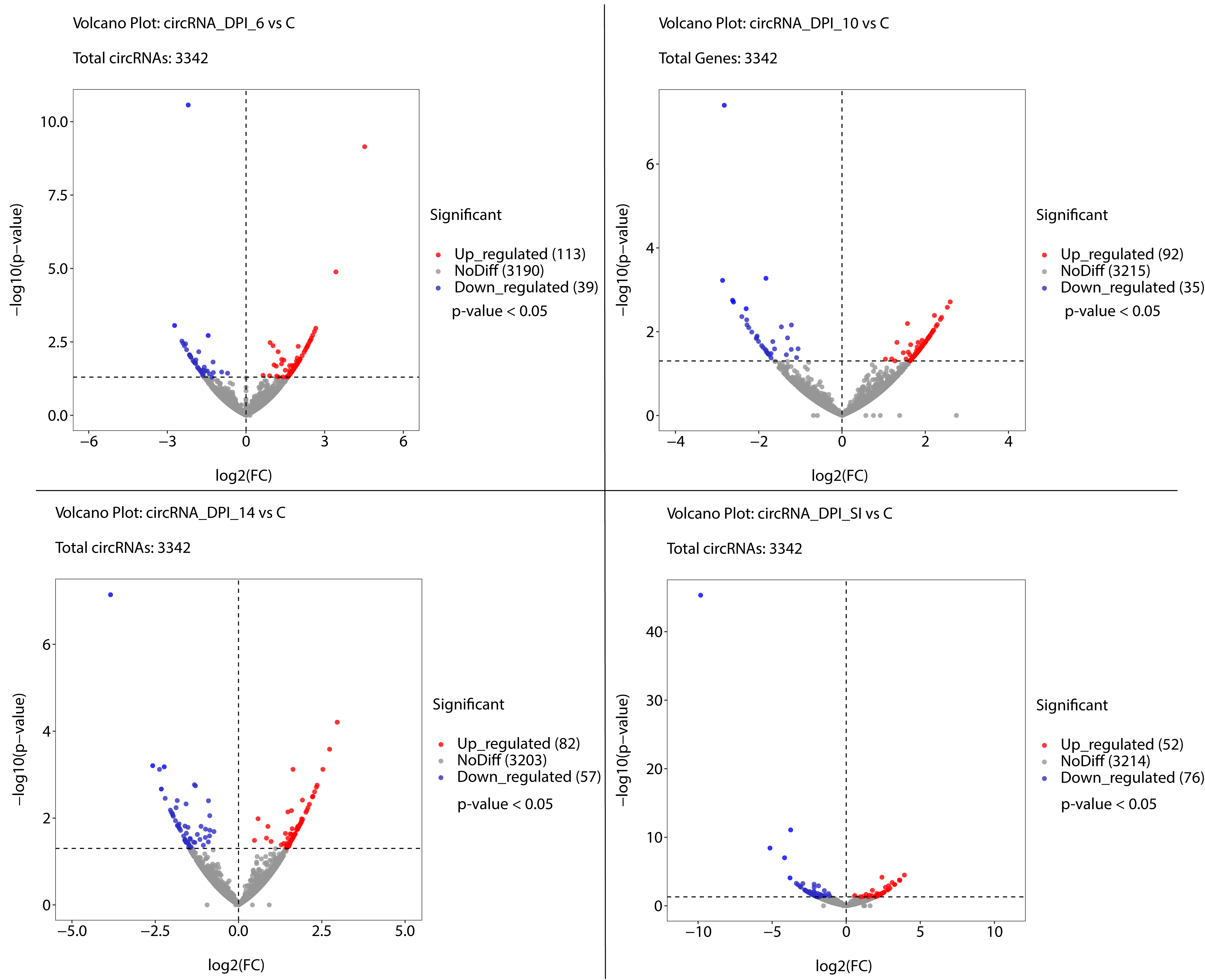

Supplement: Supplementary file 8 — Additional file 8. Volcano map of the differentially expressed (DE) circRNAs. Red represents upregulation, blue indicates downregulation, gray indicates insignificant change. The factor of P-value < 0.05 were used as the conditions for screening differential circRNAs. Total circRNAs: total number of circRNAs. [file 40249_2023_1121_MOESM8_ESM.tif]

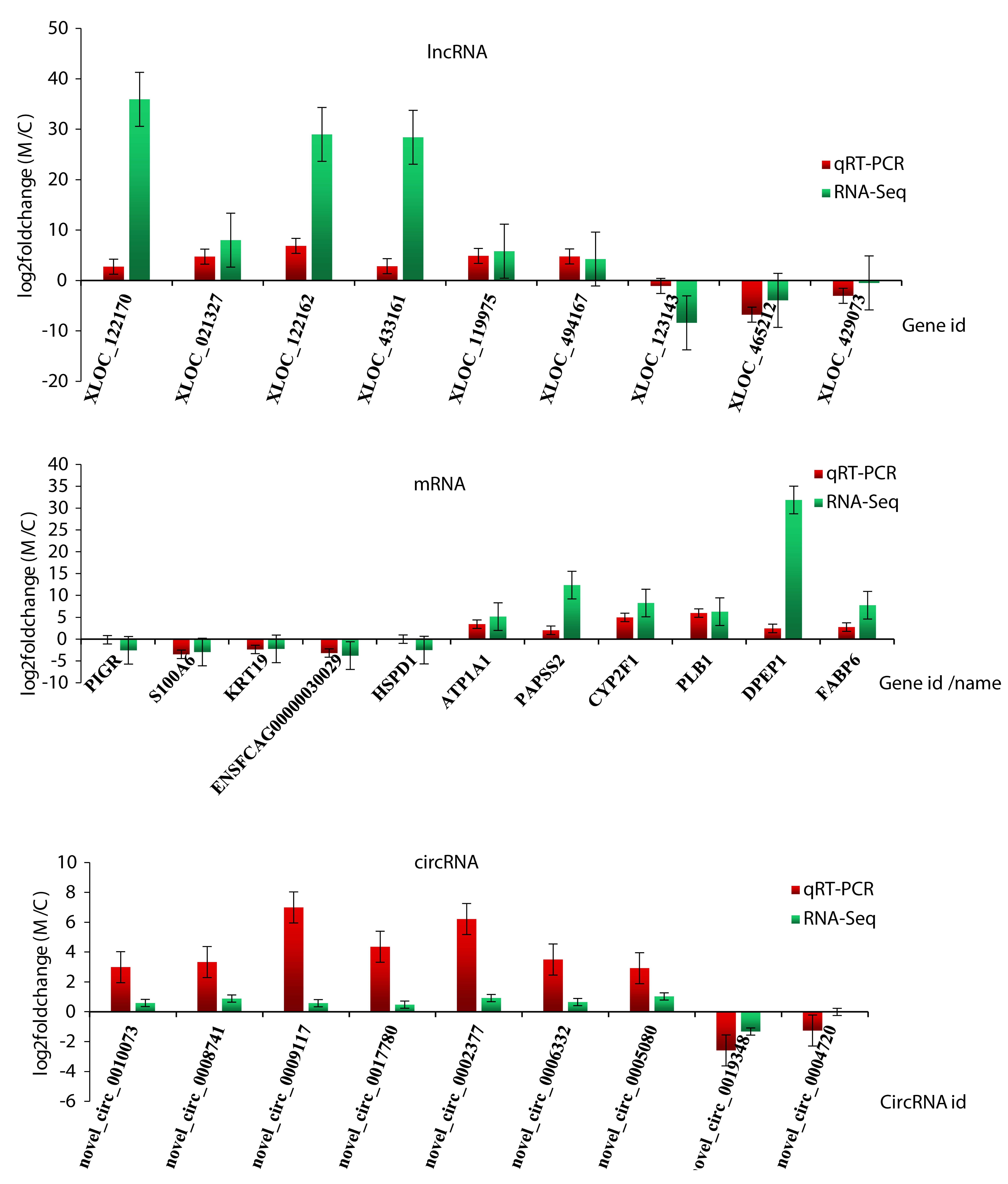

Supplement: Supplementary file 9 — Additional file 9. Verification of the RNA-seq data using qRT-PCR. qRT-PCR and RNA-Seq are represented by red, green boxes, respectively, the upper part of the x-axis represents up-regulation, and the lower part represents down-regulation. Bars represent the mean fold changes of the expression of T. gondii genes. Log2foldchange (M/C): log2foldchange (expression of infection group/expression of control group). [file 40249_2023_1121_MOESM9_ESM.tif]
